# Targeting MYC-enhanced glycolysis for the treatment of small cell lung cancer

**DOI:** 10.1186/s40170-021-00270-9

**Published:** 2021-09-23

**Authors:** Kasey R. Cargill, C. Allison Stewart, Elizabeth M. Park, Kavya Ramkumar, Carl M. Gay, Robert J. Cardnell, Qi Wang, Lixia Diao, Li Shen, You-Hong Fan, Wai Kin Chan, Philip L. Lorenzi, Trudy G. Oliver, Jing Wang, Lauren A. Byers

**Affiliations:** 1 Department of Thoracic/Head and Neck Medical Oncology, University of Texas MD Anderson Cancer Center, Houston, TX USA; 2 Department of Bioinformatics and Computational Biology, University of Texas MD Anderson Cancer Center, Houston, TX USA; 3 Department of Oncological Sciences, Huntsman Cancer Institute, University of Utah, Salt Lake City, UT USA

**Keywords:** MYC, Glycolysis, Metabolism, Small cell lung cancer, PFK158

## Abstract

**Introduction:**

The transcription factor MYC is overexpressed in 30% of small cell lung cancer (SCLC) tumors and is known to modulate the balance between two major pathways of metabolism: glycolysis and mitochondrial respiration. This duality of MYC underscores the importance of further investigation into its role in SCLC metabolism and could lead to insights into metabolic targeting approaches.

**Methods:**

We investigated differences in metabolic pathways in transcriptional and metabolomics datasets based on cMYC expression in patient and cell line samples. Metabolic pathway utilization was evaluated by flow cytometry and Seahorse extracellular flux methodology. Glycolysis inhibition was evaluated *in vitro* and *in vivo* using PFK158, a small molecular inhibitor of PFKFB3.

**Results:**

MYC-overexpressing SCLC patient samples and cell lines exhibited increased glycolysis gene expression directly mediated by MYC. Further, MYC-overexpressing cell lines displayed enhanced glycolysis consistent with the Warburg effect, while cell lines with low MYC expression appeared more reliant on oxidative metabolism. Inhibition of glycolysis with PFK158 preferentially attenuated glucose uptake, ATP production, and lactate in MYC-overexpressing cell lines. Treatment with PFK158 in xenografts delayed tumor growth and decreased glycolysis gene expression.

**Conclusions:**

Our study highlights an in-depth characterization of SCLC metabolic programming and presents glycolysis as a targetable mechanism downstream of MYC that could offer therapeutic benefit in a subset of SCLC patients.

**Supplementary Information:**

The online version contains supplementary material available at 10.1186/s40170-021-00270-9.

## Background

Small cell lung cancer (SCLC) is an aggressive neuroendocrine tumor that constitutes approximately 14% of lung cancer diagnoses [1, 2]. Current SCLC treatment options have remained largely unchanged over the past few decades. Despite recent advances, including the addition of immunotherapy in conjunction with frontline platinum-based chemotherapy for extensive stage disease, most patients still exhibit rapid relapse [3]. Thus, the 5-year survival remains low at merely 6% [2]. As such, the National Cancer Institute has designated SCLC as one of two “recalcitrant” cancers requiring advancement of treatment options [4].

SCLC characteristically exhibits loss of the tumor suppressor genes *RB1* and *TP53* [5–9]. Approximately 30% of SCLC tumors harbor amplification or overexpression of the transcription factor c*MYC* (MYC), which has been associated with more aggressive behavior and therapeutic resistance in some investigations [10–12]. Although MYC is associated with resistance, it has also been identified as a biomarker for therapeutic sensitivity to certain classes of inhibitors, including aurora kinase inhibitors [10, 13]. Even with the identification of MYC as a biomarker, presently, there are no therapeutic treatments that directly attenuate its expression [14–17]. Therefore, interventions that target pathways downstream of MYC may prove beneficial in patients with MYC-overexpressing SCLC.

One such mechanism regulated by MYC that is understudied, particularly in SCLC, is its modulation of energy metabolism. MYC plays a pivotal role in central carbon metabolism including the promotion of glycolysis and oxidative phosphorylation by supporting the balance of glucose, glutamine, and fatty acids [18]. In particular, a hallmark of cancer is an increase of aerobic glycolysis (the Warburg Effect), which enhances the uptake of glucose to produce lactate as a mechanism to generate rapid energy, nucleotides, and reducing equivalents [18–21]. This increase in glycolysis is mediated by an upregulation of genes controlling glucose transport, rate-limiting enzymes, and lactate synthesis [22], all of which can be modulated by MYC [17, 18, 23]. Although these mechanisms are beneficial for rapid growth and metastasis, some tumors are considered oxidative and utilize MYC-driven mitochondrial respiration through upregulation of genes controlling mitochondrial biogenesis, glutamine uptake, and fatty acid transport [24–26]. With the duality of MYC’s ability to control metabolic reprogramming, profiling of the energy program utilized by tumors is essential for identifying targetable mechanisms of cellular metabolism. However, to date, studies in lung cancer metabolism have primarily focused on non-small cell lung cancer (NSCLC), which constitutes roughly 85% of diagnoses [27–29], while SCLC has been left relatively unexplored. However, studies have shown that MYC-expressing SCLC is metabolically distinct and particularly vulnerable to IMPDH inhibition and arginine deprivation in preclinical settings [30, 31]. Thus, further investigation into MYC’s role in SCLC metabolism could lead to new disease insights.

Herein, we report an interrogation into the role of MYC as a driver of aerobic glycolysis in a subset of SCLC. Bimodal stratification of cell lines based on MYC protein expression support robust alterations in gene expression with approximately 40% of upregulated genes in MYC-expressing tumors and cell lines mapping to a metabolic process. Deeper investigation of the dysregulated pathways revealed an increased conversion of glucose to lactate with reduced mitochondrial oxygen utilization that can be attenuated by glycolysis inhibition in *MYC*-expressing cells. Further, analysis revealed that glycolysis inhibition delayed tumor growth in a *MYC*-expressing xenografts. Together, these data substantiate a proposal that MYC enhances glycolysis expression and stabilizes glycolytic enzymes in order to promote tumor growth. Our study highlights an in-depth characterization of SCLC metabolic programming and presents glycolysis as a targetable mechanism downstream of MYC that could offer therapeutic benefit in a subset of SCLC patients.

## Methods

### Experimental cell culture

Human SCLC cell lines H446, DMS79, H524, H526, H82, H847, H841, H196, H211, H146, SHP77, H345, H2196, H1436, H865, H1522, H1341, H1105, H1048, H1092, H1876, H69, DMS53, and H2029 were obtained from ATCC (Manassas, VA, USA) or Sigma-Aldrich (St. Louis, MO, USA). The human patient-derived xenograft (PDX) cell line NJH29 was generously provided by Dr. Julien Sage (Stanford University, Stanford, CA, USA). For experimentation, cell lines were cultured in RPMI-1640 supplemented with 10% FBS, 100 IU/mL penicillin, and 100 μg/mL streptomycin. Cells were maintained in a 37 °C humidified chamber with 5% CO_2_. All cell lines used were passaged for less than 6 months and regularly tested for *Mycoplasma* contamination using a MycoAlert Plus Kit (Lonza).

### Experimental mouse model

Six-week female athymic nude mice were used for generation of DMS79 and H446 xenograft SCLC tumor models and obtained from Envigo. One million cells were prepared in a 0.2 mL 1:1 mixture of culture media and matrigel for subcutaneous injection into the left flank of each animal then monitored daily for appearance of tumors. Two weeks following injection, animals were randomized into dosing groups when tumors reached 50 mm^3^. Animals received either vehicle or 25 mg/kg PFK158 (Selleck Chemicals) constituted in 5% DMSO, 30% PEG, 10% Tween, and 55% H_2_O by intraperitoneal (IP) injection. Animals received dosing every 2 days for a total of six treatments. Tumor volume and body weights were measured in all mice twice weekly and calculated (width^2^ × length × 0.4). A Student’s *t* test was used to determine statistical significance between vehicle- and PFK158-treated groups. All animals were maintained in accordance with the University of Texas MD Anderson Cancer Center Institutional Animal Care and Use Committee and the NIH Guide for the Care and Use of Laboratory Animals.

### Genetically engineered mouse models (GEMMs)

*Rb1*^*fl/fl*^*;Trp53*^*fl/fl*^*;MycT58A*^*LSL/LSL*^ (RPM, JAX #029971) mice were previously described [32]. Metabolomics on mouse tumors were performed as described [31]. RPM mouse lung tumor ChIP-seq data is previously described and published [31, 33]. RP cells derived from *Rb1*^*fl/fl*^*;Trp53*^*fl/fl*^ (RP) mice [34] were a generous gift from Dr. Julien Sage (Stanford University, Stanford, CA, USA). RPM-derived cells and RP-derived cells were cultured in RPMI-1640 supplemented with 10% FBS, 100 IU/mL penicillin, and 100 μg/mL streptomycin.

### Flow cytometry

One million cells were seeded in 10-cm dishes for 72 h then harvested and prepared as a single cell suspension for staining. For cells receiving treatment, 2.5 μM of PFK158 or 50 μM YN1 was applied 24 post-plating and cultured for 72 h. For surface staining, antibody was diluted in 2% FBS in PBS and incubated at 4 °C for 25 min. For intracellular staining, cells were fixed with 2% PFA at room temperature for 20 min then subjected to permeabilization (BioLegend) and staining with antibody diluted in permeabilization buffer (BioLegend) at room temperature for 20 min. For staining with metabolic dyes, the appropriate amount of dye was added to 1 mL of cell suspension and incubated at 37 °C for 10–30 min depending on the dye (see Supplementary Table 1). Cells were analyzed using either a BD LSRFortessa Flow Cytometer (BD Biosciences) or BD Accuri C6 Plus Flow Cytometer (BS Biosciences). Data analysis was done using FlowJo 10.7.1. Graphs were generated in GraphPad Prism 8, and significance was determined by a 2-way ANOVA followed by Tukey’s multiple comparisons test.

### Immunoblotting

One million cells were seeded in 10-cm dishes for 72 h then harvested and lysed with RPPA lysis buffer supplemented with protease and phosphatase inhibitor cocktail. Tumor samples were likewise prepared in RPPA lysis buffer and homogenized to lysate preparation. Lysates were centrifuged at 14,000 rpm for 10 min to remove cell debris. Total protein concentrations were measured using a DC Protein Assay Reagent (Bio-Rad). Fifty micrograms of cell lysates were boiled for 10 min at 100 °C with 2× Laemmli buffer was resolved on a 10% polyacrylamide gel and electroblotted on a nitrocellulose membrane. Membranes were blocked in 1× Caesin blocking solution (Bio-Rad) at room temperature for 1 h then incubated overnight at 4 °C with primary antibody (1:1000; see Supplementary Table 1). Membranes were washed with TBS with 0.1% Tween-20 and incubated with appropriate horseradish peroxidase-linked secondary antibodies at room temperature for 1 h. Immunoblots were visualized using the Super Signal West Pico Chemiluminescence Substrate (Thermo Fisher Scientific) on a Bio-Rad ChemiDox Touch Imaging System. Immunoblots were quantified using ImageJ and normalized to Vinculin. Where applicable, graphs were generated in GraphPad Prism 8, and significance was determined by Student’s *t* test.

### Single-agent ATP assays

SCLC cell lines were seeded at 2000 cells/well in 96-well white-bottom microtiter plates. After overnight incubation, cells were treated with PFK158 (10–0.625 μM; Selleck Chemicals) or YN1 (200–6.25 μM; Sigma-Aldrich) in one-half dilutions in triplicate for 72 h. ATP was measured by using Cell Titre Glo (Promega) and luminescence was read on a Synergy HT Microplate Reader (BioTek). IC_50_ drug concentration was estimated by modeling using R package nonlinear curve fitting as previously published (Drexplorer) [35].

### Reverse-phase proteomic array

Reverse-phase proteomic array (RPPA) was performed as previously described [36]. Antibodies against HK2, PFKFB3, and LDHA (see Supplementary Table 1) were validated using established immunoblotting approaches prior to RPPA staining.

### Apoptosis, cell viability, and proliferation analysis

Cells were seeded at one million cells/well and incubated overnight prior to treatment with 2.5 μM PFK158 for 72 h. Apoptosis was determined using FITC Annexin V Apoptosis Detection Kit with PI (BD Pharmingen) and analyzed on a BD Accuri C6 Plus. Data was generated using FlowJo 10.7.1. Cellular viability and relative proliferation after treatment with PFK158 (1.25, 2.5, or 5 μM), glucose-free media (Sigma-Aldrich), or YN1 (50 μM) were determined by 72-h treatment and trypan blue exclusion cell counting using an Invitrogen Countess Automated Cell Counter (Invitrogen) or by Live/Dead staining analyzed on a BD LSRFortessa Flow Cytometer (BD Biosciences). Graphs were generated in GraphPad Prism 8, and significance was determined by a two-way ANOVA followed by Tukey’s multiple comparisons test.

### Real-time Seahorse extracellular flux

Seahorse XFe96 FluxPak (Agilent) was hydrated overnight at 37 °C in a non-CO_2_ incubator. Seahorse XF96 cell culture microplate (Agilent) was pre-coated with 3.5 μg/cm^2^ of Cell-Tak cell and tissue adhesive for 20 min (Corning) according to manufacturer’s protocol. Lung cancer cell lines or tumor-derived cell suspension from H446 and DMS79 models in culture medium were treated with vehicle (DMSO) or PFK158 (2.5 μM) for 4 h before metabolic analysis assay. Then, 5 × 10^4^ pretreated cells were seeded in Cell-Tak pre-coated microplates and equilibrated for 45 min at 37 °C in assay media. For mitochondrial stress tests, XF RPMI pH 7.4 media (Agilent) was supplemented with 10 mM XF glucose (Agilent), 1 mM XF pyruvate (Agilent) and 2 mM XF glutamine (Agilent), and for glycolysis stress tests, XF RPMI pH 7.4 media was supplemented with 2 mM XF glutamine prior to measuring the oxygen consumption rate (OCR) and extracellular acidification rate (ECAR). Next, media were replaced with fresh assay media containing DMSO or PFK158 (2.5 μM). Seahorse XF Cell Mito Stress Test (Agilent; see Supplementary Table 3) or Seahorse XF Glycolysis Stress Test (Agilent; see Supplementary Table 3) was performed using Seahorse XFe96 Analyzer (Agilent). For Cell Mito Stress Test, after instrument calibration, OCR was measured followed by sequential injections of 1.0 μM oligomycin, 1.0 μM FCCP, and 0.5 μM rotenone and 0.5 μM antimycin A. For Cell Glycolysis Stress Test, after instrument calibration, ECAR was measured followed by sequential injections of 10 mM glucose, 1.0 μM oligomycin and 50 mM 2-DG. Data was analyzed using Seahorse Wave software 2.6. Graphs were generated in GraphPad Prism 8, and significance was determined by a one-way ANOVA followed by Sidak’s multiple comparisons test.

### Extracellular and intracellular lactate measurements

For extracellular lactate determination, cells were seeded at 5 × 10^4^ cells/well in 24-well cell culture plates and incubated overnight for attachment prior to treatment with 2.5 μM PFK158 or 50 μM YN1 for 72 h. Media supernatants were harvested and analyzed using a Lactate Plus Meter (Nova Biomedical) and Lactate Plus Meter Test Strips (Nova Biomedical). Graphs were generated in GraphPad Prism 8, and significance was determined by a two-way ANOVA followed by Tukey’s multiple comparisons test. For intracellular lactate determination, cells were seeded in triplicate at 2 × 10^4^ cells/well in a 96-well cell culture plate and incubated overnight prior to treatment with 2.5 μM PFK158 for 72 h. Cell lysates were prepared with 0.6 N HCL followed by 1 M Tris Base. Lactate concentration was determined using the Lactate-Glo Assay (Promega) by luminescence with gain adjustment normalized to a 5000 RLU maximum using a Synergy HT Microplate Reader. Using R, RLUs were converted to the log scale and correlated to *LDHA* gene expression using SCLC cell line transcriptional data [37].

### Tissue collection and histological assessment

Tumors were collected, fixed in 4% paraformaldehyde overnight, and processed in paraffin. Samples were sectioned at 4 μm and subjected to hematoxylin and eosin staining. Tissue sections were imaged using an Olympus DP80 microscope and cellSens standard software.

### Silencing RNA

Silencing RNA (siRNA) against *MYC* and *PFKFB3* (see Supplementary Table 2) were reconstituted and diluted to 10 μM in nuclease-free water. For transfection, H446, H524, and H82 cell lines were seeded at 4 × 10^5^ in 6-well cell culture plates. Lipofectamine 2000 Reagent (Invitrogen) and siRNA were diluted in Opti-MEM media (ThermoFisher Scientific) for a final concentration of 25 pmol siRNA per well. Cells were incubated for 72 h at 37 °C. ) Viability was determined by 72-h incubation and trypan blue exclusion cell counting using an Invitrogen Countess Automated Cell Counter (Invitrogen). Proliferation was determined by cell counting using an Invitrogen Countess Automated Cell Counter (Invitrogen). Glucose uptake and lactate secretion were determined as previously detailed.

### RNA sequencing of H446 tumors

Sequencing reads were aligned to human reference genome (hg38) TOPHAT2. The gene expression levels were measured by counting the mapped reads using HTSEQ. The differentially expressed genes were identified using EdgeR package. Data were transformed to log2 counts-per-million (CPM). Weakly expressed genes, whose CPM values were less than 0.5 in at least 2 samples, were filtered out. Differentially expressed genes were identified using linear model likelihood ratio and ANOVA-like tests implemented in the edgeR package. *P* values obtained from multiple binomial tests were adjusted using the false discovery rate (Benjamini–Hochberg). Significant genes were defined by a Benjamini–Hochberg–corrected *P* value cutoff of 0.05.

### Metabolite analysis

Glucose and lactate metabolites were measured in tumor tissue collected from H446 and DMS79 tumors after 28 days using Glucose-Glo and Lactate-Glo assay kits (Promega). In brief, 3 mg of flash frozen tissue was homogenized in 50 mM Tris buffer containing 8:1 0.6N HCl. After homogenization, 8:1 1M Tris buffer was added to the tissue homogenates. Samples were assayed per manufacturer’s instruction and normalized to protein expression determined by DC Protein Assay Reagent (Bio-Rad).

### Transcriptional and metabolomics datasets and statistical analyses

Transcriptional and metabolomics data from experimental datasets were publicly available [11, 37–39]. All statistic and bioinformatics analyses were performed using R unless otherwise stated. Two-sample *t* tests were used for two group comparisons. One-way ANOVA with Tukey’s multiple comparisons was used for more than two group comparison. Two-way ANOVA with Sidak’s multiple comparison was used for two group comparisons with two treatment subgroups. Spearman correlations were used for correlating genomic and proteomic measurements, as well as correlating drug screening data. In all the analyses, *P* < 0.05 was considered statistically significant.

## Results

### MYC enhances glycolytic gene expression in SCLC

**Fig. 1:**
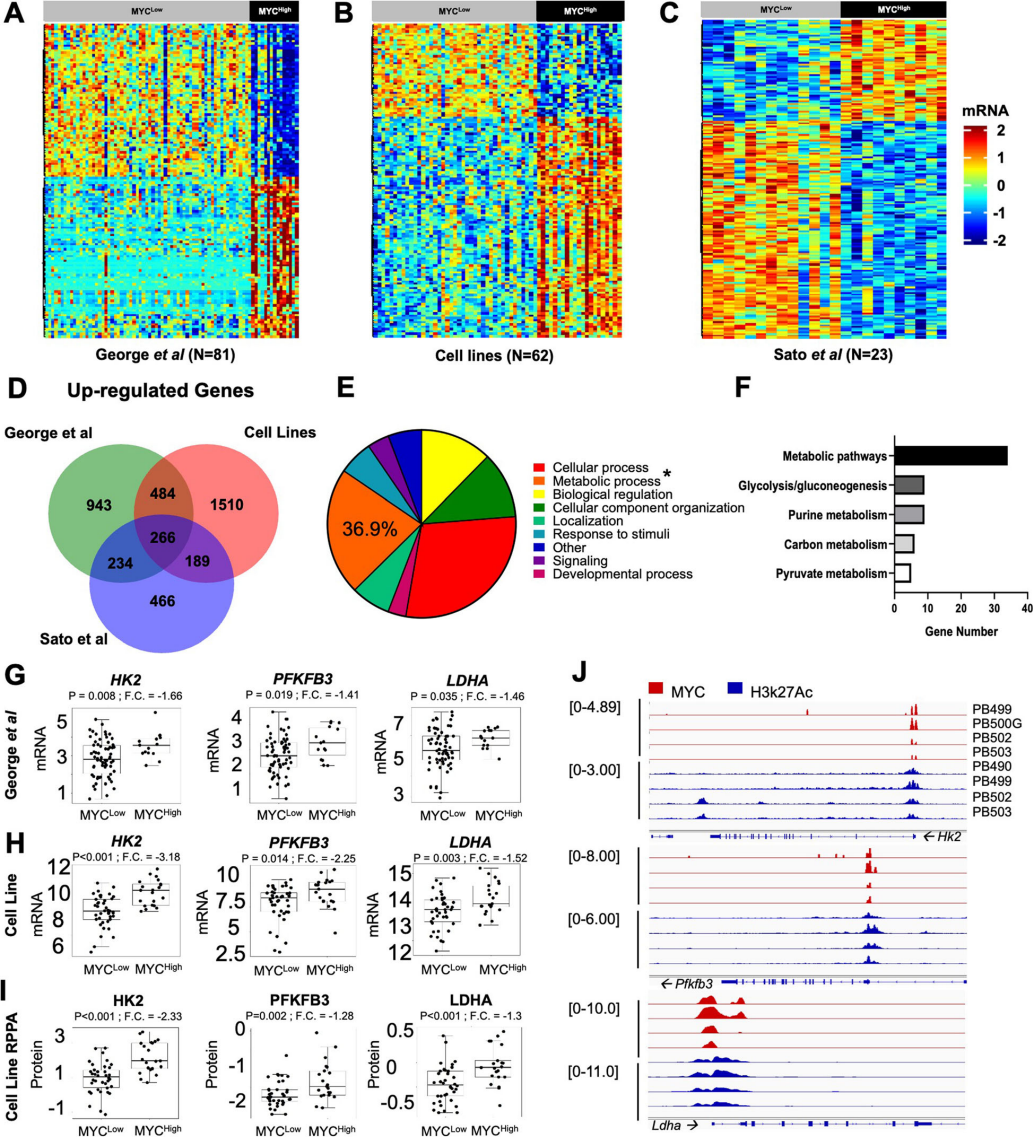
MYC directly upregulates glycolysis expression. **A–C** Bimodal *MYC* gene stratification of SCLC samples in the George et al., Gay et al., and Sato et al. datasets [11, 37, 38]. **D** Venn diagram showing the 266 upregulated gene commonalities among the three datasets. **E** Of the 266 upregulated genes, 36.9% can be linked to a metabolic process through Panther Gene Ontology Biological Pathway Analysis. **F** Similarly, several of the top GO terms generated by David Functional Enrichment Analysis are involved in central carbon and glucose metabolism. **G and H** Glycolysis genes *HK2*, *PFKFB3*, and *LDHA* exhibit increased expression in the MYC^High^ subset of patient tumors [11] and cell lines [37], respectively. **I** HK2, PFKFB3, and LDHA protein expression by RPPA is also increased in MYC^High^ cell lines. **J** ChIP-seq analysis of MYC (red) and H3K27Ac (blue) genomic binding at indicated gene loci from *N* = 4 RPM tumor samples. Arrows indicate directionality of the gene

To uncover metabolic differences promoted by *MYC*, we applied a bimodal index to both patient [11, 38] and cell line [37] SCLC transcriptome datasets to establish defined MYC subsets termed MYC^Low^ and MYC^High^ (Fig. 1a–c). Commonalities between the three datasets revealed that 266 genes were upregulated in MYC^High^ samples, and 364 genes were downregulated (*FDR* = 0.05; Fig. 1d; Supplementary Figure 1a) among all three datasets. Next, we performed gene ontology biological pathway analysis (http://pantherdb.org/) using the 266 significantly upregulated genes and found that 36.9% could be linked to a metabolic process (Fig. 1e) compared with only 19.7% of the significantly downregulated genes (Supplementary Figure 1b). Functional enrichment analysis using DAVID (https://david.ncifcrf.gov/) generated a list of KEGG pathway gene ontology (GO) terms from the significantly upregulated and downregulated genes. Five of the top GO terms for the upregulated gene list corresponded to metabolism (Fig. 1f) including metabolic pathways (34 genes), carbon metabolism (6 genes), purine metabolism (9 genes), pyruvate metabolism (5 genes), and glycolysis/gluconeogenesis (9 genes). Of the top five downregulated genes, only a single GO term (Alanine, aspartate, glutamate metabolism; 3 genes) was related to metabolism (Supplementary Figure 1c). Since many of the GO terms from the upregulated genes implicated central carbon metabolism including glycolysis, we further investigated key genes in this pathway. In both patient samples and human cell lines, the first rate-limiting gene *HK2*, the major rate-limiting gene *PFKFB3,* and the aerobic glycolysis gene *LDHA* among other glycolysis-linked genes were all significantly upregulated in MYC^High^ samples (Fig. 1g, h; Supplementary Figure 1d). Similarly, we validated the upregulation of HK2, PFKFB3, and LDHA protein expression in cell lines using reverse-phase proteomic array (RPPA), which was consistent with enhanced gene expression in the MYC^High^ subset in human-derived SCLC cell lines (Fig. 1i). Notably, these glycolysis genes were also identified as MYC target genes by chromatin immunoprecipitation sequencing (ChIP-seq) in tumors derived from the MYC-driven genetically engineered mouse model (GEMM) termed RPM (*Rb1*^*fl/fl*^*;p53*^*fl/fl*^*;MycT58A*^*LSL/LSL*^; Fig. 1j) [32]. Similarly to previous studies [31, 40], these data support that MYC mediates an increase in aerobic glycolysis by direct binding of MYC to the promoter region of regulatory glycolysis genes.

We then assessed the correlation between *MYC* expression and 225 metabolites in SCLC using data generated by the Cancer Cell Line Encyclopedia (CCLE) [39]. This analysis revealed a strong positive correlation with the amino acid proline and a negative correlation with arachidonyl carnitine (Supplementary Figure 1e-f). Notably, proline synthesis produces NAD^+^ and NADP^+^ required for glycolysis and the parallel pentose phosphate pathway [41]. Alternatively, arachidonyl carnitine is required for transport of long-chain fatty acids across the mitochondrial membrane during fatty acid oxidation [42], a process that facilities oxidative phosphorylation mechanisms. These observations of metabolite expression were expanded to RPM tumors where 125 metabolites were compared to corresponding normal mouse lung tissue. This data showed that metabolites involved in glycolysis including lactate and proline were significantly upregulated in RPM tumors, while metabolites involved in oxidative metabolism such as pyruvate and carnitine were downregulated (Supplementary Figure 1g-k).

*Inhibition of PFKFB3 suppresses ATP production and proliferation and increases apoptosis in the MYC* ^*High*^ *subset of SCLC*

**Fig. 2:**
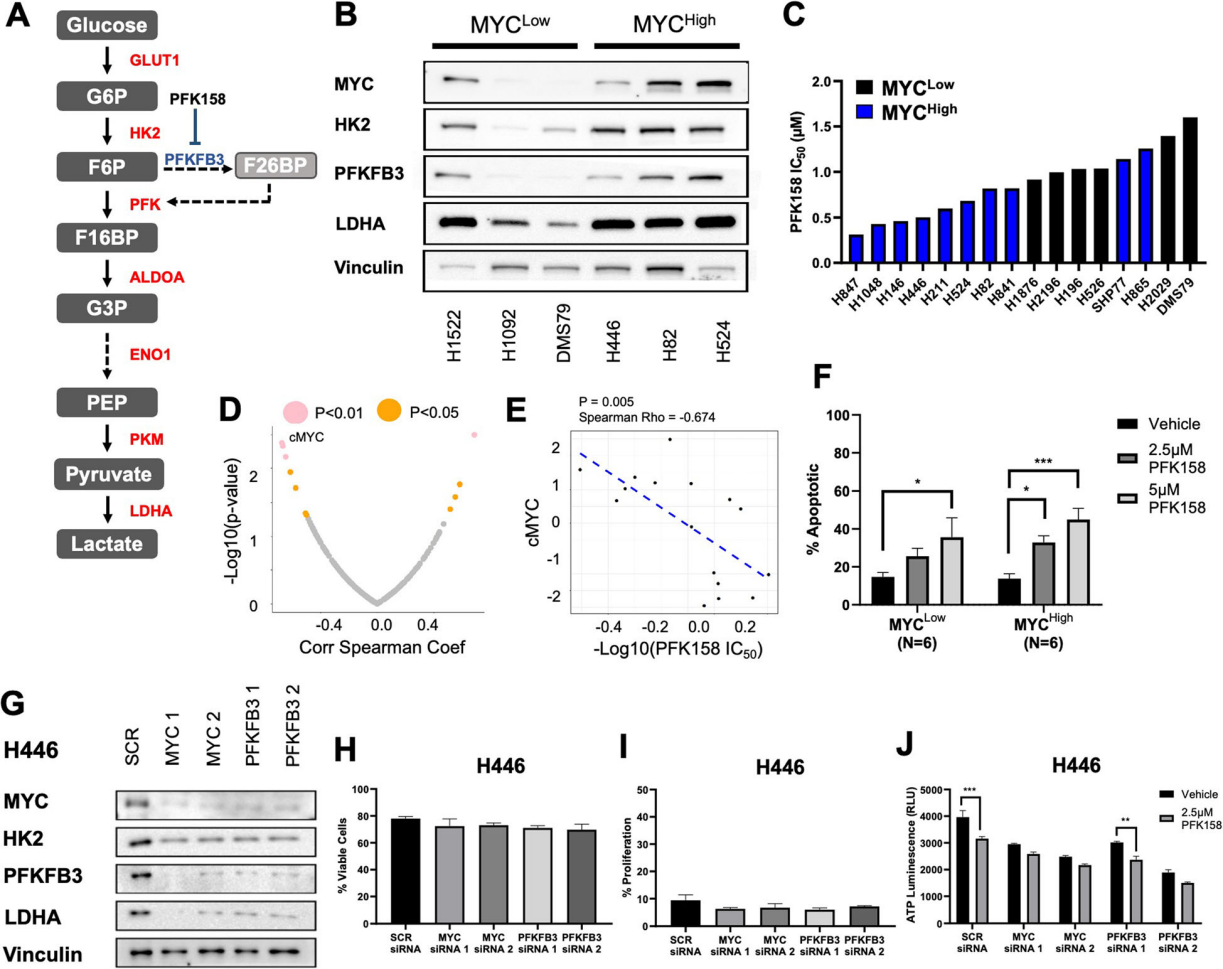
PFK158 reduces ATP production and induces apoptosis. **A** The glycolysis pathway can be inhibited at the major rate-limiting step by targeting PFKFB3 with PFK158. **B** Validation of MYC, HK2, PFKFB3, and LDHA protein expression in a panel of MYC^Low^ (H1522, H1092, DMS79) and MYC^High^ (H446, H82, H524) cell lines. **C** Calculated IC_50_ based on ATP luminescence in MYC^Low^ and MYC^High^ cell lines. **D** 11 out of 210 proteins are significantly associated with PFK158 IC_50_. **E** cMYC is more highly expressed in cell lines with lower IC_50_ values. **F** Annexin V/PI staining in MYC^Low^ cell lines (DMS79, H526, H196, H345, H2196, H1436) and MYC^High^ cell lines (H211, H847, H1930, H446, H82, H841) shows percent of apoptotic cells. **G** Immunoblots of H446 cell line transfected with SCR siRNA, MYC siRNA, and PFKFB3 siRNA showing lower MYC, HK2, PFKFB3, and LDHA protein expression with vinculin loading control. **H** The percent of viable cells among siRNA-treated H446 cells is not significantly altered. **I** The percent proliferation cells among siRNA-treated H446 cells is not significantly altered. **J** ATP luminescence of siRNA knockdown H446 cells after 24 h of 2.5 μM PFK158 treatment. (*P < 0.05; **P < 0.01; ***P < 0.005)

Given our observation of increased glycolysis in MYC^High^ samples, we confirmed MYC expression and the upregulation of the glycolysis proteins HK2, PFKFB3, and LDHA (Fig. 2a) in a panel of MYC^High^ (H446, H82, H524) human SCLC cell lines in comparison to MYC^Low^ (H1522, H1092, DMS79) cell lines (Fig. 2b). Further, previous investigations have suggested that in addition to MYC, MYCL and MYCN may play a role in mediating metabolism [31]. Therefore we confirmed that enhanced aerobic glycolysis was due to MYC expression by showing an increase in LDHA only in MYC^High^ cell lines and not cell lines classified as MYCL or MYCN (Supplementary Figure 2a).

Next, we evaluated the efficacy of the potent glycolysis inhibitor, PFK158, which is a competitive inhibitor of the regulatory glycolysis rate-limiting enzyme, PFKFB3 [43–45]. PFK158 has been investigated in a Phase I clinical trial and has demonstrated tolerability in patients [43]. We performed a dosage titration of PFK158 ranging from 0.625 to 10 μM and evaluated the IC_50_ after 72 h of treatment in six representative MYC^Low^ and 10 representative MYC^High^ cell lines (Fig. 2c). MYC^High^ cell lines were more susceptible to inhibition (MYC^Low^ IC_50_ = 1.2 μM; MYC^High^ IC_50_ = 0.7 μM; *P* = 0.009; Fig. 2c). Based on the calculated IC_50_ values, we implemented downstream biomarker analysis by correlating IC_50_ to RPPA expression levels of 210 proteins and found that 11 proteins were significantly correlated to IC_50_, with cMYC being the most significant protein as a marker of sensitivity to PFK158 (Fig. 2d). Moreover, cMYC expression was negatively correlated with IC_50_ value such that a lower IC_50_ is associated with higher cMYC (Fig. 2e). Since IC_50_ values were determined based on ATP generation and PFK158 selectively inhibits ATP-producing pathways, we also evaluated cellular viability using Annexin V/PI. Apoptotic assessment with Annexin V/PI confirmed increased cell death after treatment for 72 h with 2.5 μM and 5 μM PFK158 in six representative MYC^High^ cell lines, while the six representative MYC^Low^ cell lines showed significantly higher levels of apoptosis only after 5 μM PFK158 (Fig. 2f; Supplementary Figure 2b). PFK158-induced cell death at higher concentrations in both MYC^Low^ and MYC^High^ cell lines is not entirely unsurprising since it is not specific for aerobic glycolysis and will inhibit both the fermentation and oxidation of glucose; however, our data indicate that its effects are greater in MYC-expressing cells that are dependent on glucose. We also evaluated apoptosis in MYC non-expressing GEMM (*Rb1*^*fl/fl*^*; p53*^*fl/fl*^ termed RP) and RPM-derived cell lines and found that treatment with PFK158 lead to similar levels of apoptosis in both models at 2.5 μM; however, at 5 μM RP, cells exhibited an average of 40% apoptosis, while RPM cells averaged 80% apoptosis (Supplementary Figure 2c). These data indicate that the calculated IC_50_ is not solely indicative of cell death, and instead ATP reduction is an output of decreased metabolic function. Therefore, based on these data, we used 2.5 μM PFK158 for all downstream analysis.

Next, to determine the effect of glycolysis inhibition on proliferation, we stained both human SCLC cell lines and RP/RPM GEMM cell lines with violet proliferation dye then treated with PFK158 for 72 h and found significantly reduced proliferation exclusively in MYC^High^ cells treated with PFK158 (Supplementary Figure 2d-e). Further, MYC^High^ cells cultured in glucose-free media for 72 h also showed a significant reduction in both viability and proliferation (Supplementary Figure 2f-g). Collectively, these data indicate that PFK158 may selectively induce cell death in the MYC^High^ subset by decreasing the available ATP, nucleotides, and amino acids required for proliferation and cellular survival.

Next, we addressed whether knockdown of *MYC* and *PFKFB3* resulted in lower glycolysis protein expression or altered viability and proliferation using siRNA. Indeed, we revealed marked decreases in MYC, HK2, PFKFB3, and LDHA in H446 cells treated with siRNA against both MYC and PFKFB3 for 72 h (Fig. 2g). Although surprising, PFKFB3 knockdown also lead to reduced MYC expression, which we hypothesized is due to a negative feedback mechanism previously described by Berg et al. [46]. We did not observe any significant differences in cellular viability or proliferation after knockdown (Fig. 2h–i; Supplementary Figure 2g). Lastly, after 72-h treatment with siRNA, H446 cells were subjected to 24-h treatment with 2.5 μM PFK158 then analyzed for ATP content. Significant decreases in ATP was observed between control siRNA (SCR) treated with vehicle and PFK158 as well as in one PFKFB3 siRNA treated with vehicle and PFK158 (Fig. 2j). Similar results were obtained with H524 and H82 cell lines (Supplementary Figure 2h-m). Importantly, these data indicate that both *MYC* and *PFKFB3* are necessary for glycolysis protein expression, and without their expression, cells are less susceptible to PFK158.

### MYC enhances glycolysis while suppressing mitochondrial function, which can be modulated by PFK158 in SCLC

**Fig. 3:**
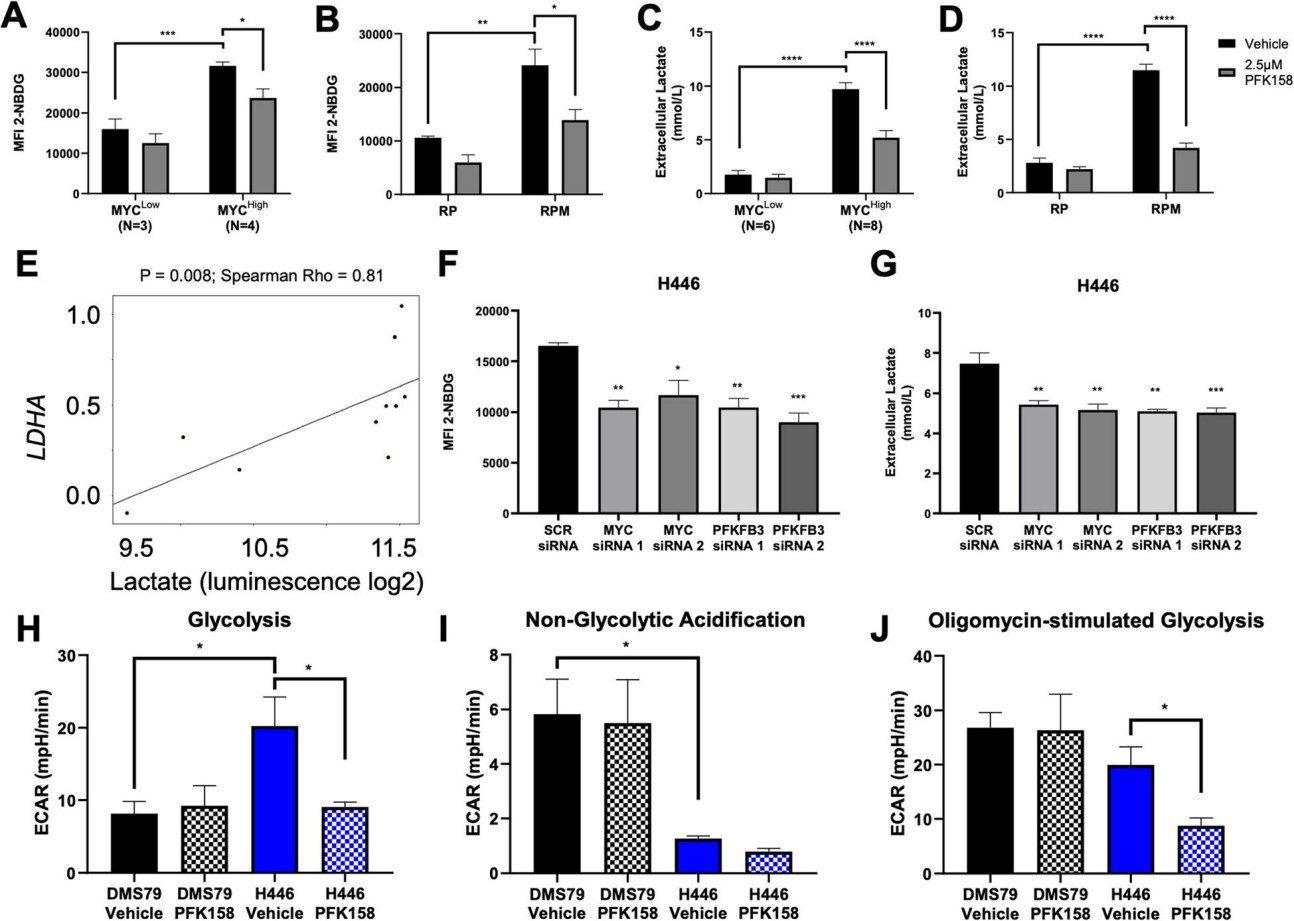
MYC^High^ cells are more glycolytic and sensitive to glycolysis inhibition. **A** Glucose uptake is higher in MYC^High^ (H82, H446, H1048, H847) cell lines compared with MYC^Low^ (DMS79, H345, H196, cell lines and is significantly decreased with PFK158 treatment. **B** RPM cells have higher glucose uptake compared with RP cells and is reduced with PFK158 treatment. **C** Similarly, extracellular lactate is higher in MYC^High^ (H1930, H847, H524, H841, H446, H146, NJH29, H1048) cell lines compared with MYC^Low^ (DMS79, H196, H526, H345, H2196, H1105) cell lines and reduced with PFK158 treatment. **D** Extracellular lactate is higher in RPM cell lines and reduced with PFK158 treatment. **E** Intracellular lactate significantly and positively correlates with LDHA expression (cell lines: NJH29, H1092, H446, H1436, H82, H1930, DMS79, H1048, H841, H526). **F** Glucose uptake in significantly lower in H446 cells treated with siRNA against *MYC* and *PFKFB3* compared with a SCR negative control. **G** Extracellular lactate is significantly reduced in H446 cells treated with siRNA against *MYC* and *PFKFB3* compared with a SCR negative control. **H** Based on the ECAR, glycolysis is significantly higher in the representative MYC^High^ cell line H446 as compared with the representative MYC^Low^ cell line DMS79. There were no changes in glycolysis in DMS79 cell treated with PFK158; however, glycolysis was significantly decreased in H446 cells treated with PFK158. **I** Acid produced by non-glycolytic pathways was significantly higher in DMS79 cells regardless of treatment. **J** The level of oligomycin-stimulated glycolysis indicative of forced glycolytic utilization is significantly lower in PFK158-treated H446 cells (*P < 0.05; **P < 0.01; ***P < 0.005; ****P < 0.001)

It is well established that MYC controls many metabolic pathways that become dysregulated in cancer including glycolysis and lactate production [18]. Since PFK158 leads to reduced ATP generation in MYC^High^ cell lines, we anticipated that glucose uptake and lactate secretion would be decreased in the presence of drug. Indeed, a panel of four MYC^High^ cell lines and RPM cells treated with 2.5 μM PFK158 for 4 h then incubated with the fluorescent glucose analogue 2-NBDG exhibited a significant decrease in glucose uptake (Fig. 3a, b). Conversely, a panel of three MYC^Low^ cell lines and RP cells initially took in significantly less glucose, which was not reduced in the presence of PFK158 (Fig. 3a, b). Similarly, lactate excreted into culture medium was significantly higher in eight MYC^High^ and RPM cells compared with six MYC^Low^ and RP cell lines, which was greatly reduced after 72-h treatment with PFK158 (Fig. 3c, d). In addition to extracellular lactate, we also measured intracellular lactate in MYC^Low^ and MYC^High^ cell lines. There were no differences in intracellular lactate between four MYC^Low^ cell lines at baseline and PFK158 treatment; however, there is a downward trend between six MYC^High^ cell lines at baseline PFK158 treatment (Fig. 3e). The insignificant difference in intracellular lactate levels between MYC^Low^ and MYC^High^ cells is likely due to the reuptake of lactate, enhanced lactate shuttling, or interconversion between pyruvate/lactate for utilization of an alternative carbon source in mitochondrial metabolism, as often seen in predominantly oxidative cancers such as NSCLC [47–49]. Although intracellular lactate levels were not significantly altered, there was direct correlation between intracellular lactate and *LDHA* gene expression (Fig. 3e). Additionally, we validated these data using siRNA against *MYC* and *PFKFB3*, which we showed exhibited downregulation of glycolysis proteins in H446, H524, and H82 cell lines (Fig. 2g). Glucose uptake and extracellular lactate were both significantly decreased among *MYC* and *PFKFB3* siRNAs compared with the SCR control (Fig. 3f, g; Supplementary Figure 3b-e). Using an additional glycolysis inhibitor (YN1), we also evaluated IC_50_ values (MYC^Low^ IC_50_ = 12.4 μM; MYC^High^ IC_50_ = 6.8 μM; Supplementary Figure 3f), glucose uptake, and lactate secretion. Although there was no significant difference in glucose uptake, lactate levels were significantly reduced after treatment in MYC^High^ cell lines (Supplementary Figure 3g-h). These data support that MYC influences the rate of aerobic glycolysis through mediation of pathway-specific regulatory enzymes.

Next, we tested whether the glycolytic rate in MYC^High^ cells was higher than in MYC^Low^ cells. We selected DMS79 as a representative MYC^Low^ SCLC cell line and H446 as a representative MYC^High^ SCLC cell line. To explore glycolysis, we performed a glycolysis stress test using Seahorse extracellular flux analysis to measure the extracellular acidification rate (ECAR), which is an indicator of a glucose to lactate glycolytic conversion. This assay is done in the presence of a series of substrates and inhibitors including glucose (glycolysis substrate), oligomycin (ATP synthase inhibitor), and 2-DG (glycolysis inhibitor) (Supplementary Table 3). As anticipated, glycolysis was highest in H446 cells without PFK158 inhibitor, but significantly decreased when glycolysis was inhibited (Fig. 3h). DMS79 cells displayed low levels of glycolysis regardless of inhibition but had a high rate of non-glycolytic acidification (Fig. 3h, i). Interestingly, DMS79 cells with and without inhibitor were able to transition to glycolysis utilization after administration of oligomycin, a potent mitochondrial inhibitor (Fig. 3j). Conversely, H446 cells treated with PFK158 were unable to efficiently utilize glycolysis even when ATP synthase was inhibited (Fig. 3j), indicating that PFK158 substantially inhibits glycolysis in MYC^High^ cells and their ability to metabolically reprogram to glucose-independent pathways as a mechanism to evade cell death.

**Fig. 4:**
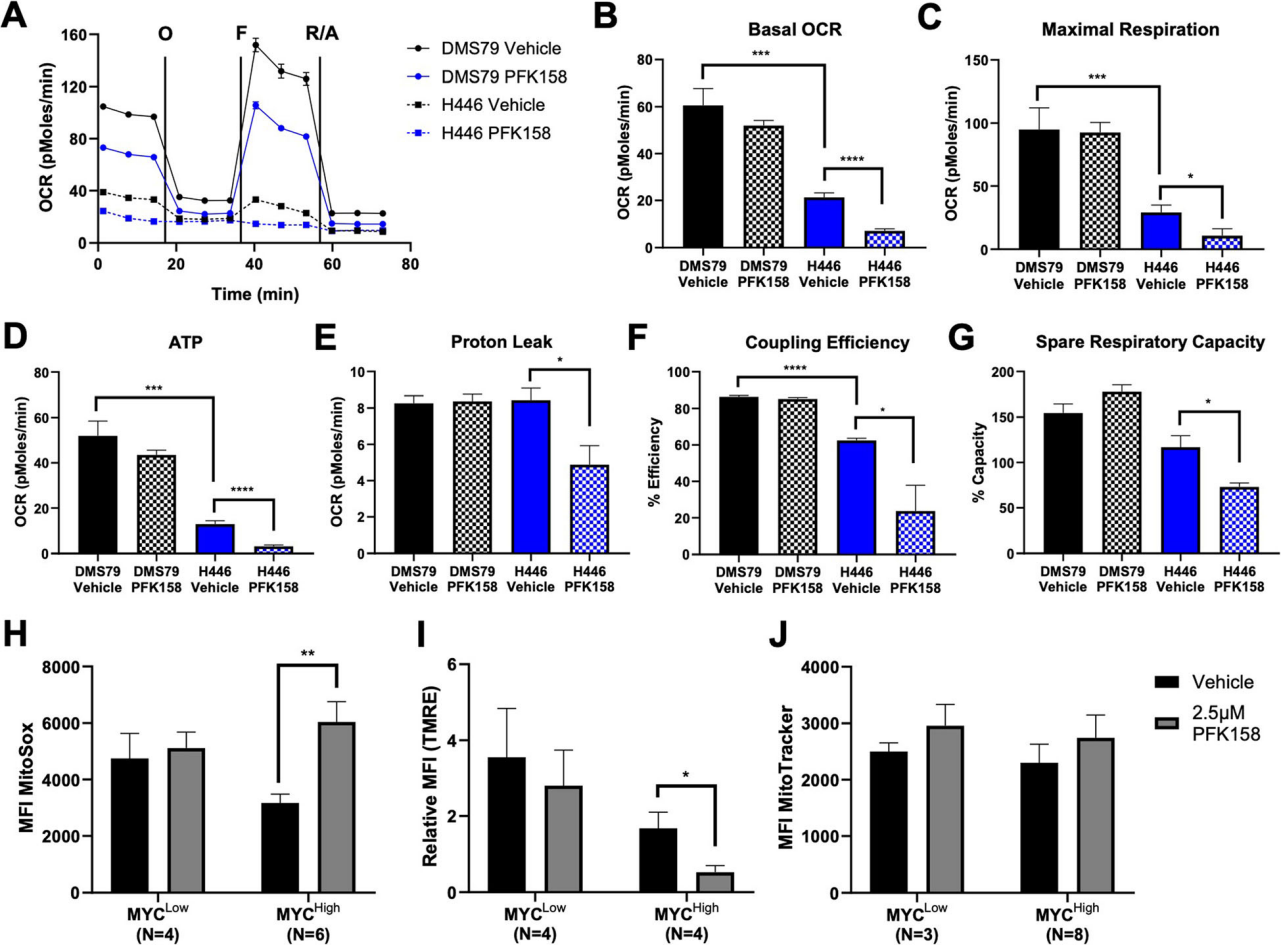
**PFK158-treated MYC**^**High**^**cells do not increase oxidative respiration mechanisms. A** Seahorse extracellular flux mito stress test monitored the oxygen consumption rate after the administration of oligomycin, FCCP, and rotenone and antimycin A. **B** Basal oxygen consumption is significantly reduced in H446 cells compared with DMS79. In H446 cells treated with PFK158, basal oxygen consumption is also significantly reduced compared with untreated cells. **C** Maximal oxygen consumption is significantly reduced in H446 cells compared with DMS79. In H446 cells treated with PFK158, maximal oxygen consumption is also significantly reduced compared with untreated cells. **D** Mitochondria-generated ATP is significantly reduced in H446 cells compared with DMS79. In H446 cells treated with PFK158, ATP is also significantly reduced based on untreated cells. **E** Proton leak is unchanged between DMS79 and H446 cells, but significantly reduced in H446 cells treated with PFK158. **F** Coupling efficiency is significantly reduced in H446 cells compared with DMS79. In H446 cells treated with PFK158, coupling efficiency is also significantly reduced compared with untreated cells. **G** Spare respiratory capacity is unchanged between DMS79 and H446 cells, but significantly reduced in H446 cells treated with PFK158. **H** Reactive oxygen species (ROS) generation is significantly increased in MYC^High^ (H446, H524, H146, H211, H1048, H841) cells treated with PFK158 compared with MYC^Low^ (DMS79, H196, H526, H345) cells. **I** The mitochondrial membrane potential is significantly reduced in MYC^High^ (H446, H524, H146, H211) cells treated with PFK158 compared with MYC^Low^ (DMS79, H196, H526, H345) cells. **J** There are no differences in mitochondrial content regardless of MYC status or treatment; MYC^Low^ cell lines: DMS79, H196, H526; MYC^High^ cell lines: H446, H524, H146, H211, H1048, H841, H82, H865 (*P < 0.05; **P < 0.01; ***P < 0.005; ****P < 0.001)

To determine whether the effects of PFK158 disrupt mitochondrial function, we also performed robust mitochondrial assessment. In addition to extracellular acidification, Seahorse XFe96 analyzers monitor the oxygen consumption rate (OCR) through a mitochondrial stress test which administers the substrates oligomycin, FCCP (mitochondrial membrane uncoupler), and rotenone/antimycin A (electron transport chain complex I/III inhibitors) (Supplementary Table 3). DMS79 cells were unaffected by PFK158 treatment with respect to OCR (Fig. 4a, b); however, vehicle-treated DMS79 cells had significantly higher basal OCR, maximal respiration, ATP, and coupling efficiency compared to H446 vehicle-treated cells (Fig. 4b–d, f). As for PFK158-treated H446 cells, treatment resulted in significantly reduced basal OCR, maximal respiration, ATP, proton leakage, coupling efficiency, and spare respiratory capacity (Fig. 4b–g). Since these mitochondrial output assays are lower in MYC-expressing cells at baseline and further repressed after PFK158 treatment; these data indicate that glucose is the primary carbon source utilized by MYC^High^ SCLC. However, the continued pattern of reduced oxygen consumption suggests that these cells cannot metabolically reprogram towards an oxidative phenotype in order to continue supporting cellular proliferation.

In line with this data, we next investigated mitochondrial health and density using flow cytometry. Cells were treated with PFK158 for 72 h prior to incubation with MitoSox (reactive oxygen species indicator), TMRE (mitochondrial membrane potential indicator), or MitoTracker (mitochondrial content dye). ROS are small-molecule free radicals that are often upregulated during periods of cellular stress [50, 51]. Treatment with PFK158 significantly increased the levels of ROS produced by six MYC^High^ cell lines (Fig. 4h); however, there was no significant difference in ROS production between four MYC^Low^- and six MYC^High^-treated cell lines (*P* = 0.46; Fig. 4h). Although elevated ROS levels can serve as signaling molecules to promote cellular proliferation and cancer metastasis, an overabundance will trigger cell death mechanisms including apoptosis [51], which occurs after treatment with PFK158 in MYC^High^ cell lines due to insufficient ATP generation and reduced flux through oxidative respiration pathways. Another measurement of mitochondrial health is the membrane potential where a lower membrane potential often correlates with higher levels of ROS [52]. Indeed, mitochondrial membrane potential measured by TMRE was significantly lower in four MYC^High^ cell lines treated with PFK158 (Fig. 4i). Lastly, we determined the density of mitochondria across three MYC^Low^- and eight MYC^High^-treated and -untreated cell lines, and no significant differences were noted regardless of treatment status (Fig. 4j). Collectively, our data demonstrate that cells expressing MYC preferentially utilize aerobic glycolysis, while those that do not efficiently rely on oxidative respiration. Moreover, PFK158 treatment is highly effective at inhibiting aerobic glycolysis and renders cells that are dependent on this pathway incapable of generating sufficient energy.

### PFK158 delays tumor growth in MYC-expressing xenografts

**Fig. 5:**
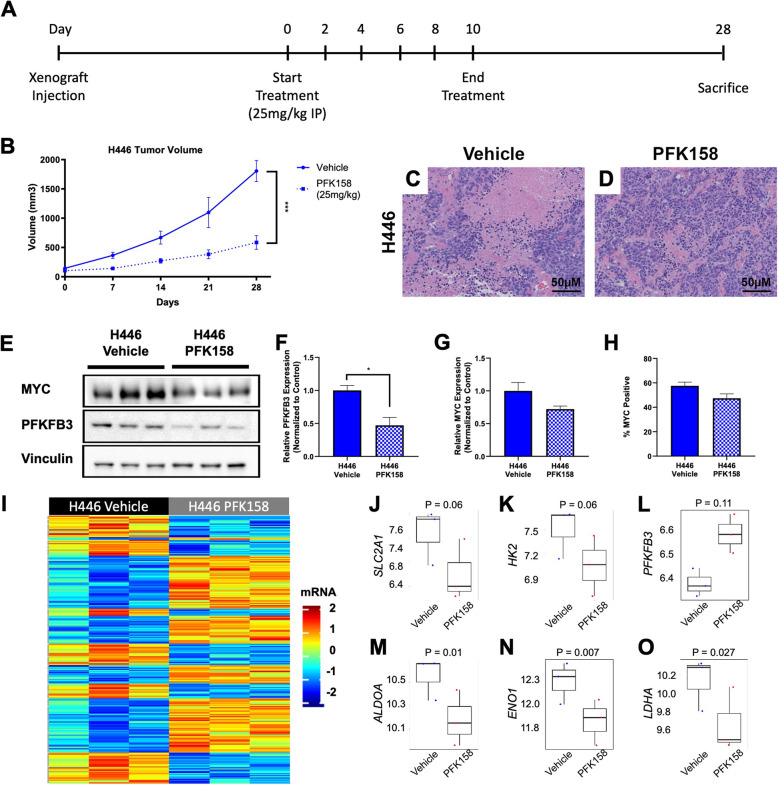
PFK158 reduces tumor growth in a MYC^High^ xenograft model. **A** H446 xenografts were given 25 mg/kg PFK158 by intraperitoneal injection every other day for 10 days. **B** PFK158-treated animals exhibited a significant delay in tumor growth. **C** and **D** H&E staining showing that PFK158-treated animals have less necrotic tissue. **E** Immunoblot against MYC and PFKFB3 with loading control Vinculin. **F** PFKFB3 expression was significantly reduced in H446 tumors from animals treated with PFK158. **G** and **H** MYC expression was lower but not significantly so in tumors regardless of PFK158 treatment when evaluated by immunoblotting or flow cytometry, respectively. **I** Heatmap showing transcriptional expression changes between H446 tumors treated with vehicle or PFK158. **J–L** *SLC2A1*, *HK2*, and *PFKFB3* gene expression are not significantly altered following PFK158 treatment. **M–O** Downstream glycolysis enzyme genes *ALDOA*, *ENO1*, and *LDHA* are significantly lower following PFK158 treatment. (*P < 0.05; ***P < 0.005)

To investigate the effects of glycolysis inhibition on *in vivo* tumor growth, we derived xenograft models from the MYC^Low^ human cell line DMS79 (*N* = 19) and the MYC^High^ human cell line H446 (*N* = 19). Once tumors were visible, mice were randomized into either vehicle (*N* = 10) or PFK158 (25 mg/kg; *N* = 9) treatment groups. Treatments were given every other day for the duration of 10 days then monitored for tumor growth until day 28 or upon a tumor volume of 2000 mm^3^ (Fig. 5a). PFK158 significantly delayed tumor growth even after cessation of treatment on day 10 in H446 tumors (Fig. 5b). In line with these observed growth differences, hematoxylin & eosin (H&E) staining of tumors treated with PFK158 exhibited reduced necrosis; likely due to the slower rate of growth compared with vehicle-treated tumors, which rapidly progressed (Fig. 5c, d). A significant tumor growth difference was not observed in DMS79 tumors nor were there notable histological differences (Supplementary Figure 4a-c). Consistent with previous reports, no toxicity was observed due to PFK158 treatment (Supplementary Figure 4a; Supplementary Figure 4f).

Next, we evaluated protein expression by immunoblotting against MYC and PFKFB3 (Fig. 5e). PFK158 significantly decreased PFKFB3 expression (Fig. 5f) consistent with previous reports [44]. While there was no statistically significant decrease in MYC expression in H446 tumors treated with PFK158 when evaluated by immunoblotting and flow cytometry (Fig. 5g, h), there is a noticeable reduction that is similar to that seen in our *in vitro* work (Fig. 2g; Supplementary Figure 2h&k). We also performed RNA-sequencing on vehicle and PFK158-treated tumors and identified more than 2000 significantly altered genes (Fig. 5i). We performed biological pathway analysis on the significantly dysregulated genes, 17.9% of which were linked to a metabolic process (Supplementary Figure 4f). Moreover, several of the top dysregulated KEGG pathways implicated GO terms that are directly related to metabolism, cellular proliferation, cell cycle, and cell death among others (Supplementary Figure 4g). Interestingly, glycolysis genes that code for enzymes early in the glycolysis pathway (SLC2A1, HK2, PFKFB3) are not significantly altered (Fig. 5j–l), which is consistent with a higher trending glucose-to-lactate ratio in H446 PFK158-treated tumors (*P* = 0.09; Supplementary Figure 4h-i). Genes that are involved after PFKFB3 (ALDOA, ENO1, LDHA) are significantly downregulated (Fig. 5m–o). Lastly, we performed Seahorse extracellular flux analysis on H446 tumors treated with vehicle or PFK158 and found that the tumors treated with PFK158 have a reduced basal and maximal oxygen consumption (OCR) and lower baseline rate of glycolysis (ECAR; Supplementary Figure 4J-K). Although Seahorse extracellular flux was performed on a single tumor analyzed in six technical replicates, these data further suggest that PFK158 decreases aerobic glycolysis *in vivo*.

## Discussion

It is widely accepted that a subset of SCLC frequently exhibits MYC amplification or overexpression [32, 53, 54]. Although the role of MYC in driving glycolysis and cancer progression is well documented [18, 31, 40, 55], it has yet to be successfully targeted for therapeutic benefit [16]. Our previous studies show that MYC defines a subset of SCLC that is comprised largely of the SCLC-N, SCLC-P, and SCLC-I subtypes, while SCLC-A remains predominantly low in MYC expression [10, 32, 33, 37]. While it is plausible that the mechanisms driving a particular metabolic phenotype may also be regulated by the subtype-specific transcription factors (*ASLC1*, *NEUROD1*, *POU2F3*), teasing out the nuances of these pathways beyond glycolysis and oxidative respiration may be challenging. Notably, however, the present study is the first to define the metabolic program utilized in the broader MYC-expressing subset and provide direct evidence to support the use of metabolic modulators for treatment in SCLC. In particular, MYC^High^ cell lines and tumors proved vulnerable to glycolysis inhibition, while MYC^Low^ remained largely unresponsive. These findings indicate that MYC contributes to the use of aerobic glycolysis and acts as a driver of high aggressive clinical SCLC. Moreover, the absence of MYC promotes oxidative mechanisms. These data illustrate that MYC defines the predominant metabolic pathway utilized by tumors such that MYC-expressing tumors exhibit a classic Warburg effect phenotype, while MYC non-expressing tumors have a strong reliance on mitochondrial respiration.

Although MYC promotes rapid cellular growth leading to tumor metastasis, the interaction between the tumor and its microenvironment often contributes to drug resistance and subsequent relapse. Our study reveals that glucose is the major substrate for MYC-expressing SCLC cell lines. As such, increased influx of glucose contributes to higher rates of lactate secreted and recycled by adjacent tumor cells. Of note, T-cells require nutrients including glucose for activation and effector function in the tumor microenvironment (TME) [56]. Both glucose uptake and lactate secretion by the tumor serve to inhibit immune cell function by (1) reducing the available glucose supply contributing to T-cell dysfunction [57] and (2) generating lactate leading to an acidic, immunosuppressive environment [58]. It is postulated, however, that inhibition of glycolysis would be selective for the cells that have the highest metabolic demand with limited effect on terminally differentiated cells [59, 60]. This suggests that targeting glycolysis, which is preferentially upregulated in MYC-expressing cells, can mitigate an acidic TME while restoring nutrient balance. This would facilitate immune infiltration, and further investigation is needed to provide a basis for implementation of metabolic inhibition in combination with immunotherapy for SCLC treatment.

Despite recent advances in standard-of-care by combining platinum-based chemotherapy with immunotherapy, the 5-year survival rate of SCLC remains low [3, 61]. Further, interrogation into metabolic modulation for the treatment of cancer is readily explored; however, our understanding of the contribution of metabolic alterations to SCLC is relatively understudied. Our investigation demonstrates a unique clinical opportunity for targeting cellular metabolism in this subset of disease. Although the present work identifies metabolism as a promising area for clinical development, the future direction of this study requires additional *in vivo* characterization and would benefit to utilize spontaneous SCLC models (including RP and RPM GEMMs) to determine the efficacy of using MYC as a selective biomarker of metabolism and treatment response. Additionally, it has been shown that cisplatin-based chemotherapy resistance is associated with an oxidative phenotype, while carboplatin-based chemotherapy resistance selects for glycolytic cells [62–65]. While metabolic selectivity of chemotherapy has been shown, there are currently no metabolically-based treatments available to SCLC patients. Future biomarker analysis for resistance to chemotherapy may shed light on potential combinatorial approaches to combat frontline resistance. While there is limited data from clinical trials involving PFK158 (NCT02044861), further investigations into the metabolic profile of platinum-based chemotherapy resistance are required in order for combinatorial approaches to be applied in SCLC.

## Conclusion

In summary, these findings highlight an MYC-driven SCLC subset with a unique vulnerability to glycolytic inhibition. The present study remains focused on the role of glycolysis in promoting rapid cell growth leading to aggressive metastasis in patients, however underscores the importance of metabolic programming for both glycolytic and oxidative pathways. Despite the aggression of MYC-driven SCLC, this population only comprises approximately 30% of all SCLC diagnoses. Therefore, further interrogations into the use of oxidative phosphorylation inhibitors in tumors and cell lines that do not express MYC are required to fully understand the potential for metabolic intervention as a therapeutic strategy among all SCLC subtypes.

## Supplementary Information

**Additional file 1: Supplementary Table 1**: List of antibodies. Comprehensive list of all antibodies, dyes, and fluorescent analogues utilized in this investigation. **Supplementary Table 2**: List of siRNA. Comprehensive list of the siRNA utilized in this investigation. **Supplementary Table 3**: Seahorse extracellular flux reagents. Assay kit reagents used for the glycolysis stress test and mito stress test during Seahorse extracellular flux analysis. **Supplementary Figure 1**: Metabolic characterization of MYC-expressing tumors and cell lines. A) There are 364 genes commonly down regulated in the MYC^High^ subset of the George et al, Gay et al, and Sato et al datasets [11, 37, 38]. B) 19.7% of the down regulated genes are linked to a metabolic process. C) The top GO terms for the down regulated genes are not implicated in metabolic pathways. D) *HK2*, *PFKFB3*, and *LDHA* gene expression are decreased in MYC^High^ patient sample s[38]. E) Proline significantly and positively correlates to *MYC* expressio n[39]. F) Arachidonyl-carnitine significantly and negatively correlates to *MYC* expressio n[39]. G) Metabolites isolated from normal lung tissue and spontaneous RPM lung tumors revealed 57 out of 108 significantly altered metabolites. H-I) Lactate and proline are significantly increased in RPM tumors. J-K) Pyruvate and carnitine are significantly reduced in RPM tumors. **Supplementary Figure 2**: PFK158 and glucose restriction reduce viability and proliferation. A) Immunoblot of cell lines classified as MYC, MYCL, or MYCN showing protein expression of MYC and LDHA with HSP90 loading control. B) Representative MYC^Low^ (DMS79) and MYC^High^ (H446) apoptotic flow plots after treatment with 2.5μM PFK158. C) The percent of apoptotic RP and RPM cells increases with higher doses of PFK158. D) The percent proliferation of RP and RPM cells is significantly decreased after 2.5μM PFK158 treatment. E) The percent proliferation of MYC^Low^ (H2029, DMS79, H526) and MYC^High^ (H847, H146, H82, H446) cells is significantly decreased after 2.5μM PFK158 treatment. F-G) Glucose restriction reduces viability and proliferation in MYC^High^ (H146, H446, H847, H865) cell lines but not MYC^Low^ (H526, H2029, DMS79, H1836) cell lines. H) Immunoblots of H524 cell line transfected with SCR siRNA, MYC siRNA, and PFKFB3 siRNA showing lower MYC, HK2, PFKFB3, and LDHA protein expression with vinculin loading control. I) The percent of viable cells among siRNA treated H524 cells is not significantly altered. J) The percent proliferation cells among siRNA treated H524 cells is not significantly altered. K) Immunoblots of H82 cell line transfected with SCR siRNA, MYC siRNA, and PFKFB3 siRNA showing lower MYC, HK2, PFKFB3, and LDHA protein expression with vinculin loading control. L) The percent of viable cells among siRNA treated H82 cells is not significantly altered. M) The percent proliferation cells among siRNA treated H82 cells is not significantly altered. (*P<0.05; **P<0.01; ****P<0.001). **Supplementary Figure 3**: Targeting glycolysis reduces ATP generation and lactate secretion. A) Intracellular lactate trends downward in MYC^High^ (NJH29, H446, H82, H1930, H1048, H841) and MYC^Low^ (H1092, H1436, DMS79, H526) cells treated with 2.5μM PFK158. B) Glucose uptake in significantly lower in H524 cells treated with siRNA against *MYC* and *PFKFB3* compared to a SCR control. C) Extracellular lactate is significantly reduced in H524 cells treated with siRNA against *MYC* and *PFKFB3* compared to a SCR control. D) Glucose uptake in significantly lower in H82 cells treated with siRNA against *MYC* and *PFKFB3* compared to a SCR control. E) Extracellular lactate is significantly reduced in H82 cells treated with siRNA against *MYC* and *PFKFB3* compared to a SCR control. F) IC_50_ values of cell lines treated with the glycolysis inhibitor YN1. G) There are no significant differences in glucose uptake after YN1 treatment; MYC^Low^ (H1436, DMS79, H1092, H526, H1522) cell lines; MYC^High^ (H446, H82, NJH29, H841, H524) cell lines. H) Extracellular lactate is significantly reduced after YN1 treatment in MYC^High^ cell lines; MYC^Low^ (H1436, DMS79, H1092, H526, H1522) cell lines; MYC^High^ (H446, H82, NJH29, H841, H524) cell lines. (*P<0.05; **P<0.01; ***P<0.005; ****P<0.001). **Supplementary Figure 4**: PFK158 treatment in H446 and DMS79 tumors. A) DMS79 tumor growth curve showing no statistical difference. B-C) H&E of DMS79 tumors showing similar pathology. D-E) Body weight curves of DMS79 and H446 xenografts with no statistical differences. F) 17.9% of genes that are significantly altered between vehicle and PFK148-treated H446 xenografts are linked to a metabolic process. G) Several of the top GO terms related to the significantly altered genes between the vehicle and PFK158-treated H446 animals. H-I) The glucose-to-lactate metabolite ratio in H446 and DMS79 tumors. J) Oxygen consumption rate of H446 xenograft tumors (*N=*1) treated with vehicle or PFK158 analyzed in six technical replicates. K) Extracellular acidification rate of H446 xenograft tumors (*N=*1) treated with vehicle or PFK158 analyzed in six technical replicates.

## Data Availability

Publically available datasets are cited where appropriate. Data generated during this study is not yet available, but can be deposited into a publically available repository upon publication, if necessary.

## Abbreviations

2-DG: 2-Deoxy-D-glucose
2-NBDG: 2-(*N*-(7-Nitrobenz-2-oxa-1,3- diazol -4-yl)Amino)-2- Deoxy-glucose
ALDOA: Aldolase fructose-bisphosphate A
ATP: Adenosine triphosphate
CCLE: Cancer cell line encyclopedia
CO_2_: Carbon dioxide
DAVID: Database for Annotation, Visualization, and Integrate Discovery
DMSO: Dimethyl sulfoxide
ECAR: Extracellular acidification rate
ENO1: Enolase 1
FBS: Fetal bovine serum
FCCP: Trifluoromethoxy carbonylcyanide phenylhydrazone
GEMM(s): Genetically engineered mouse model(s)
GO: Gene ontology
H&E: Hematoxylin and eosin
HK2: Hexokinase 2
IC_50_: Half-maximal inhibitory concentration
IMPDH: Inosine-5-monophosphate dehydrogenase
KEGG: Kyoto Encyclopedia of Genes and Genomes
LDHA: Lactate dehydrogenase
MYC/cMYC: MYC proto-oncogene
NAD: Nicotinamide adenine dinucleotide
NADP: Nicotinamide adenine dinucleotide phosphate
NSCLC: Non-small cell lung cancer
OCR: Oxygen consumption rate
Opti-MEM: Optimal minimum essential media
PFA: Paraformaldehyde
PFK158: 6-phosphofructo-2-kinase/ fructose-2,6-bisphosphatases isoform 3 inhibitor PFK158
PFKFB3: 6-phosphofructo-2-kinase/ fructose-2,6-bisphosphatases isoform 3
PI: Propidium iodide
ROS: Reactive oxygen species
RB1: Retinoblastoma 1
RP: *Rb1* ^*fl/fl*^ *;p53* ^*fl/fl*^
RPM: *Rb1* ^*fl/fl*^ *;p53* ^*fl/fl*^ *;MycT58A* ^*LSL/LSL*^
RPMI: Roswell Park Memorial Institute cell culture media
RPPA: Reverse phase protein array
SCLC: Small cell lung cancer
Sirna: Silencing RNA
SLC2A1: Solute carrier family 2 member 1 (GLUT1)
TME: Tumor microenvironment
TMRE: Tetramethylrhodamine, ethyl ester
TP53: P53 tumor suppressor
YN1: 7,8-Dihydroxy-3-(4-hydroxyphenyl)-4H-chromen-4-one, 6-Phosphofructo-2-kinase/fructose-2,6-bisphosphatase 3 Inhibitor

## References

[1] N H, AM N, M K, D M, A B, M Y (2019). SEER Cancer Statistics Review (CSR) 1975-2016.

[2] Society AC (2019). Cancer Facts and Figures 2019.

[3] Horn L, Mansfield AS, Szczesna A, Havel L, Krzakowski M, Hochmair MJ (2018). First-line atezolizumab plus chemotherapy in extensive-stage small-cell lung cancer. N Engl J Med.

[4] Institute NC (2014). Scientific framework for small cell lung cancer (SCLC).

[5] Inamura K (2017). Lung cancer: understanding its molecular pathology and the 2015 WHO classification. Front Oncol.

[6] Park KS, Liang MC, Raiser DM, Zamponi R, Roach RR, Curtis SJ, Walton Z, Schaffer BE, Roake CM, Zmoos AF, Kriegel C, Wong KK, Sage J, Kim CF (2011). Characterization of the cell of origin for small cell lung cancer. Cell Cycle.

[7] Wildey G, Dowlati A (2016). Genomic alterations in small cell lung cancer and their clinical relevance. Transl Lung Cancer Res.

[8] Takahashi T, Takahashi T, Suzuki HI, Hida T, Sekido Y, Ariyoshi Y (1991). The p53 gene is very frequently mutated in small-cell lung cancer with a distinct nucleotide substitution pattern. Oncogene..

[9] Peifer M, Fernandez-Cuesta L, Sos ML, George J, Seidel D, Kasper LH (2012). Integrative genome analyses identify key somatic driver mutations of small-cell lung cancer. Nat Genet.

[10] Cardnell RJ (2017). li L, Sen T, Bara R, Tong P, Fujimoto J, et al. Protein expression of TTF1 and cMYC define distinct molecular subgroups of small cell lung cancer with unique vulnerabilities to Aurora kinase inhibition, DLL3 targeting, and other targeted therapies. Oncotarget..

[11] George J, Lim JS, Jang SJ, Cun Y, Ozretic L, Kong G (2015). Comprehensive genomic profiles of small cell lung cancer. Nature..

[12] Rudin CM, Poirier JT, Byers LA, Dive C, Dowlati A, George J, Heymach JV, Johnson JE, Lehman JM, MacPherson D, Massion PP, Minna JD, Oliver TG, Quaranta V, Sage J, Thomas RK, Vakoc CR, Gazdar AF (2019). Molecular subtypes of small cell lung cancer: a synthesis of human and mouse model data. Nat Rev Cancer.

[13] Owonikoko TK, Niu H, Nackaerts K, Csoszi T, Ostoros G, Mark Z, Baik C, Joy AA, Chouaid C, Jaime JC, Kolek V, Majem M, Roubec J, Santos ES, Chiang AC, Speranza G, Belani CP, Chiappori A, Patel MR, Czebe K, Byers L, Bahamon B, Li C, Sheldon-Waniga E, Kong EF, Williams M, Badola S, Shin H, Bedford L, Ecsedy JA, Bryant M, Jones S, Simmons J, Leonard EJ, Ullmann CD, Spigel DR, C14018 study investigators (2020). Randomized phase II study of paclitaxel plus alisertib versus paclitaxel plus placebo as second-line therapy for SCLC: primary and correlative biomarker analyses. J Thorac Oncol.

[14] Miller DM, Thomas SD, Islam A, Muench D, Sedoris K (2012). c-Myc and cancer metabolism. Clin Cancer Res.

[15] Gabay M, Li Y, Felsher DW. MYC activation is a hallmark of cancer initiation and maintenance. Cold Spring Harb Perspect Med. 2014;4(6):1-13. 10.1101/cshperspect.a014241. 10.1101/cshperspect.a014241 PMC4031954 24890832

[16] Chen H, Liu H, Qing G (2018). Targeting oncogenic Myc as a strategy for cancer treatment. Signal Transduct Target Ther.

[17] Dang CV (2012). MYC on the path to cancer. Cell..

[18] Goetzman ES, Prochownik EV (2018). The role for Myc in coordinating glycolysis, oxidative phosphorylation, glutaminolysis, and fatty acid metabolism in normal and neoplastic tissues. Front Endocrinol (Lausanne).

[19] Feron O (2009). Pyruvate into lactate and back: from the Warburg effect to symbiotic energy fuel exchange in cancer cells. Radiother Oncol.

[20] Seyfried TN, Shelton LM (2010). Cancer as a metabolic disease. Nutr Metab (Lond).

[21] Warburg OH (1925). The Metabolism of Carcinoma Cells. Cancer Res.

[22] Li XB, Gu JD, Zhou QH (2015). Review of aerobic glycolysis and its key enzymes - new targets for lung cancer therapy. Thorac Cancer.

[23] Dong Y, Tu R, Liu H, Qing G (2020). Regulation of cancer cell metabolism: oncogenic MYC in the driver's seat. Signal Transduct Target Ther.

[24] Camarda R, Zhou AY, Kohnz RA, Balakrishnan S, Mahieu C, Anderton B, Eyob H, Kajimura S, Tward A, Krings G, Nomura DK, Goga A (2016). Inhibition of fatty acid oxidation as a therapy for MYC-overexpressing triple-negative breast cancer. Nat Med.

[25] Wise DR, Thompson CB (2010). Glutamine addiction: a new therapeutic target in cancer. Trends Biochem Sci.

[26] Marengo B, Garbarino O, Speciale A, Monteleone L, Traverso N, Domenicotti C (2019). MYC expression and metabolic redox changes in cancer cells: a synergy able to induce chemoresistance. Oxidative Med Cell Longev.

[27] Chen PH, Cai L, Huffman K, Yang C, Kim J, Faubert B, et al. Metabolic diversity in human non-small cell lung cancer cells. Mol Cell. 2019;76(5):838-851.e5. 10.1016/j.molcel.2019.08.028. 10.1016/j.molcel.2019.08.028 PMC6898782 31564558

[28] Hensley CT, Faubert B, Yuan Q, Lev-Cohain N, Jin E, Kim J, Jiang L, Ko B, Skelton R, Loudat L, Wodzak M, Klimko C, McMillan E, Butt Y, Ni M, Oliver D, Torrealba J, Malloy CR, Kernstine K, Lenkinski RE, DeBerardinis RJ (2016). Metabolic heterogeneity in human lung tumors. Cell..

[29] Zappa C, Mousa SA (2016). Non-small cell lung cancer: current treatment and future advances. Transl Lung Cancer Res.

[30] Huang F, Ni M, Chalishazar MD, Huffman KE, Kim J, Cai L, Shi X, Cai F, Zacharias LG, Ireland AS, Li K, Gu W, Kaushik AK, Liu X, Gazdar AF, Oliver TG, Minna JD, Hu Z, DeBerardinis RJ (2018). Inosine monophosphate dehydrogenase dependence in a subset of small cell lung cancers. Cell Metab.

[31] Chalishazar MD, Wait SJ, Huang F, Ireland AS, Mukhopadhyay A, Lee Y, Schuman SS, Guthrie MR, Berrett KC, Vahrenkamp JM, Hu Z, Kudla M, Modzelewska K, Wang G, Ingolia NT, Gertz J, Lum DH, Cosulich SC, Bomalaski JS, DeBerardinis RJ, Oliver TG (2019). MYC-driven small-cell lung cancer is metabolically distinct and vulnerable to arginine depletion. Clin Cancer Res.

[32] Mollaoglu G, Guthrie MR, Bohm S, Bragelmann J, Can I, Ballieu PM (2017). MYC drives progression of small cell lung cancer to a variant neuroendocrine subtype with vulnerability to Aurora kinase inhibition. Cancer Cell.

[33] Ireland AS, Micinski AM, Kastner DW, Guo B, Wait SJ, Spainhower KB, et al. MYC drives temporal evolution of small cell lung cancer subtypes by reprogramming neuroendocrine fate. Cancer Cell. 2020;38(1):60-78.e12. 10.1016/j.ccell.2020.05.001. 10.1016/j.ccell.2020.05.001 PMC7393942 32473656

[34] Meuwissen R, Linn SC, Linnoila RL, Zevenhoven J, Mooi WJ, Berns A (2003). Induction of small cell lung cancer by somatic inactivation of both Trp53 and Rb1 in a conditional mouse model. Cancer Cell.

[35] Tong P, Coombes KR, Johnson FM, Byers LA, Diao L, Liu DD, Lee JJ, Heymach JV, Wang J (2015). drexplorer: a tool to explore dose-response relationships and drug-drug interactions. Bioinformatics..

[36] Cardnell RJ, Feng Y, Mukherjee S, Diao L, Tong P, Stewart CA, Masrorpour F, Fan YH, Nilsson M, Shen Y, Heymach JV, Wang J, Byers LA (2016). Activation of the PI3K/mTOR pathway following PARP inhibition in small cell lung cancer. PLoS One.

[37] Gay CM, Stewart CA, Park EM, Diao L, Groves SM, Heeke S, et al. Patterns of transcription factor programs and immune pathway activation define four major subtypes of SCLC with distinct therapeutic vulnerabilities. Cancer Cell. 2021;39(3):346-360.e7. 10.1016/j.ccell.2020.12.014. 10.1016/j.ccell.2020.12.014 PMC8143037 33482121

[38] Sato T, Kaneda A, Tsuji S, Isagawa T, Yamamoto S, Fujita T, Yamanaka R, Tanaka Y, Nukiwa T, Marquez VE, Ishikawa Y, Ichinose M, Aburatani H (2013). PRC2 overexpression and PRC2-target gene repression relating to poorer prognosis in small cell lung cancer. Sci Rep.

[39] Li H, Ning S, Ghandi M, Kryukov GV, Gopal S, Deik A, Souza A, Pierce K, Keskula P, Hernandez D, Ann J, Shkoza D, Apfel V, Zou Y, Vazquez F, Barretina J, Pagliarini RA, Galli GG, Root DE, Hahn WC, Tsherniak A, Giannakis M, Schreiber SL, Clish CB, Garraway LA, Sellers WR (2019). The landscape of cancer cell line metabolism. Nat Med.

[40] Kim JW, Zeller KI, Wang Y, Jegga AG, Aronow BJ, O'Donnell KA, Dang CV (2004). Evaluation of myc E-box phylogenetic footprints in glycolytic genes by chromatin immunoprecipitation assays. Mol Cell Biol.

[41] D'Aniello C, Patriarca EJ, Phang JM, Minchiotti G (2020). Proline metabolism in tumor growth and metastatic progression. Front Oncol.

[42] Longo N, Frigeni M, Pasquali M (2016). Carnitine transport and fatty acid oxidation. Biochim Biophys Acta.

[43] Redman RA, Pohlmann PR, Kurman MR, Tapolsky G, Chesney JA (2015). A phase I, dose-escalation, multi-center study of PFK-158 in patients with advanced solid malignancies explores a first-in-man inhbibitor of glycolysis. J Clin Oncol.

[44] Sarkar Bhattacharya S, Thirusangu P, Jin L, Roy D, Jung D, Xiao Y, Staub J, Roy B, Molina JR, Shridhar V (2019). PFKFB3 inhibition reprograms malignant pleural mesothelioma to nutrient stress-induced macropinocytosis and ER stress as independent binary adaptive responses. Cell Death Dis.

[45] Modal S, Roy D, Bhattacharya S, Jin L, Jung D, Zhang S (2019). Therapeutic targeting of PFKFB3 with a novel glycolyticinhibitor PFK158 promotes lipophagy and chemosensitivity ingynecologic cancers. Int J Cancer.

[46] Berg JM, Tymoczko JL, Stryer L. The glycolytic pathway is tightly controlled. New York 2002 5th edition:[Available from: https://www.ncbi.nlm.nih.gov/books/NBK22395/.

[47] Ying M, Guo C, Hu X (2019). The quantitative relationship between isotopic and net contributions of lactate and glucose to the tricarboxylic acid (TCA) cycle. J Biol Chem.

[48] Faubert B, Li KY, Cai L, Hensley CT, Kim J, Zacharias LG, Yang C, Do QN, Doucette S, Burguete D, Li H, Huet G, Yuan Q, Wigal T, Butt Y, Ni M, Torrealba J, Oliver D, Lenkinski RE, Malloy CR, Wachsmann JW, Young JD, Kernstine K, DeBerardinis RJ (2017). Lactate metabolism in human lung tumors. Cell..

[49] Majem B, Nadal E, Munoz-Pinedo C. Exploiting metabolic vulnerabilities of non small cell lung carcinoma. Semin Cell Dev Biol. 2019;98:54-62. 10.1016/j.semcdb.2019.06.004. 10.1016/j.semcdb.2019.06.004 31238096

[50] Liou GY, Storz P (2010). Reactive oxygen species in cancer. Free Radic Res.

[51] Perillo B, Di Donato M, Pezone A, Di Zazzo E, Giovannelli P, Galasso G (2020). ROS in cancer therapy: the bright side of the moon. Exp Mol Med.

[52] Suski JM, Lebiedzinska M, Bonora M, Pinton P, Duszynski J, Wieckowski MR (2012). Relation between mitochondrial membrane potential and ROS formation. Mitochondrial Bioenergetics.

[53] Gazdar A, Carney D, Nau M, Minna J (1985). Characterization of variant subclasses of cell lines derived from small cell lung cancer having distinctive biochemical, morphological, and growth properties. Cancer Res.

[54] Johnson BE, Ihde DC, Muakuch RW, Gazdar AF, Carney DN, Oie H (1987). Myc family oncogene amplification in tumor cell lines established from small cell lung cancer patients and its relationship to clinical status and course. J Clin Invest.

[55] Huang F, Huffman KE, Wang Z, Wang X, Li K, Cai F, et al. Guanosine triphosphate links MYC-dependent metabolic and ribosome programs in small-cell lung cancer. J Clin Invest. 2021;131(1):1-18. 10.1172/JCI139929. 10.1172/JCI139929 PMC7773395 33079728

[56] Ho PC, Bihuniak JD, Macintyre AN, Staron M, Liu X, Amezquita R, Tsui YC, Cui G, Micevic G, Perales JC, Kleinstein SH, Abel ED, Insogna KL, Feske S, Locasale JW, Bosenberg MW, Rathmell JC, Kaech SM (2015). Phosphoenolpyruvate is a metabolic checkpoint of anti-tumor T cell responses. Cell..

[57] Chang CH, Qiu J, O'Sullivan D, Buck MD, Noguchi T, Curtis JD (2015). Metabolic competition in the tumor microenvironment is a driver of cancer progression. Cell..

[58] Fischer K, Hoffmann P, Voelkl S, Meidenbauer N, Ammer J, Edinger M, Gottfried E, Schwarz S, Rothe G, Hoves S, Renner K, Timischl B, Mackensen A, Kunz-Schughart L, Andreesen R, Krause SW, Kreutz M (2007). Inhibitory effect of tumor cell-derived lactic acid on human T cells. Blood..

[59] Bettencourt IA, Powell JD (2017). Targeting metabolism as a novel therapeutic approach to autoimmunity, inflammation, and transplantation. J Immunol.

[60] Patel CH, Leone RD, Horton MR, Powell JD (2019). Targeting metabolism to regulate immune responses in autoimmunity and cancer. Nat Rev Drug Discov.

[61] Antonia SJ, López-Martin JA, Bendell J, Ott PA, Taylor M, Eder JP, Jäger D, Pietanza MC, le DT, de Braud F, Morse MA, Ascierto PA, Horn L, Amin A, Pillai RN, Evans J, Chau I, Bono P, Atmaca A, Sharma P, Harbison CT, Lin CS, Christensen O, Calvo E (2016). Nivolumab alone and nivolumab plus ipilimumab in recurrent small-cell lung cancer (CheckMate 032): a multicentre, open-label, phase 1/2 trial. Lancet Oncol.

[62] Zaal EA, Berkers CR (2018). The influence of metabolism on drug response in cancer. Front Oncol.

[63] Wangpaichitr M, Wu C, Li YY, Nguyen DJM, Kandemir H, Shah S, Chen S, Feun LG, Prince JS, Kuo MT, Savaraj N (2017). Exploiting ROS and metabolic differences to kill cisplatin resistant lung cancer. Oncotarget..

[64] Sullivan EJ, Kurtoglu M, Brenneman R, Liu H, Lampidis TJ (2014). Targeting cisplatin-resistant human tumor cells with metabolic inhibitors. Cancer Chemother Pharmacol.

[65] Liu Y, He C, Huang X (2017). Metformin partially reverses the carboplatin-resistance in NSCLC by inhibiting glucose metabolism. Oncotarget..

