# Supplementary material for: Targeting MYC-enhanced glycolysis for the treatment of small cell lung cancer
Source: Cancer Metab. 2021 Sep 23;9:33. doi: 10.1186/s40170-021-00270-9 (PMC8461854; doi:10.1186/s40170-021-00270-9)
Supplement: Supplementary file 1 — Additional file 1: Supplementary Table 1: List of antibodies. Comprehensive list of all antibodies, dyes, and fluorescent analogues utilized in this investigation. Supplementary Table 2: List of siRNA. Comprehensive list of the siRNA utilized in this investigation. Supplementary Table 3: Seahorse extracellular flux reagents. Assay kit reagents used for the glycolysis stress test and mito stress test during Seahorse extracellular flux analysis. Supplementary Figure 1: Metabolic characterization of MYC-expressing tumors and cell lines. A) There are 364 genes commonly down regulated in the MYCHigh subset of the George et al, Gay et al, and Sato et al datasets [11, 37, 38]. B) 19.7% of the down regulated genes are linked to a metabolic process. C) The top GO terms for the down regulated genes are not implicated in metabolic pathways. D) HK2, PFKFB3, and LDHA gene expression are decreased in MYCHigh patient sample s[38]. E) Proline significantly and positively correlates to MYC expressio n[39]. F) Arachidonyl-carnitine significantly and negatively correlates to MYC expressio n[39]. G) Metabolites isolated from normal lung tissue and spontaneous RPM lung tumors revealed 57 out of 108 significantly altered metabolites. H-I) Lactate and proline are significantly increased in RPM tumors. J-K) Pyruvate and carnitine are significantly reduced in RPM tumors. Supplementary Figure 2: PFK158 and glucose restriction reduce viability and proliferation. A) Immunoblot of cell lines classified as MYC, MYCL, or MYCN showing protein expression of MYC and LDHA with HSP90 loading control. B) Representative MYCLow (DMS79) and MYCHigh (H446) apoptotic flow plots after treatment with 2.5μM PFK158. C) The percent of apoptotic RP and RPM cells increases with higher doses of PFK158. D) The percent proliferation of RP and RPM cells is significantly decreased after 2.5μM PFK158 treatment. E) The percent proliferation of MYCLow (H2029, DMS79, H526) and MYCHigh (H847, H146, H82, H446) cells is signi [file 40170_2021_270_MOESM1_ESM.docx]

| **Antibody/Dye/Analogue** | **Protocol/purpose** | **Final concentration** | **Incubation (min)** | **Company** | **Catalog No.** |
| --- | --- | --- | --- | --- | --- |
| **MYC** | Intracellular staining | 1:50 | 20 | Cell Signaling | 12855S |
| **MitoSox** | ROS | 5µM | 15 | ThermoFisher | M36008 |
| **MitoTracker** | Mitochondria | 200nM | 30 | ThermoFisher | M7514 |
| **TMRE** | Membrane potential | 150nM | 15 | BD Pharmingen | 564696 |
| **2-NBDG** | Glucose uptake | 100µM | 10 | PeproTech | 1860768 |
| **Annexin V/PI** | Apoptosis | 5µL/5µL | 15 | BioLegend | 640914 |
| **Live/Dead Stain** | Viability | 1:300 | 5 | BD Horizon | 565694 |
| **Hexokinase 2** | Immunoblotting | 1:1000 | Overnight | Abcam | AB228819 |
| **PFKFB3** | Immunoblotting | 1:1000 | Overnight | Cell Signaling | 3582S |
| **MYC** | Immunoblotting | 1:1000 | Overnight | Cell Signaling | 5605S |
| **LDHA** | Immunoblotting | 1:1000 | Overnight | Cell Signaling | 13123S |
| **Vinculin** | Immunoblotting | 1:1000 | Overnight | Sigma | V9131 |

**Supplementary Table 1: List of antibodies.** Comprehensive list of all antibodies, dyes, and fluorescent analogues utilized in this investigation.

| **siRNA** | **Name** | **Transcript type** | **Assay ID** | **Company** | **Catelog No.** |
| --- | --- | --- | --- | --- | --- |
| **Control** | SCR siRNA | *Silencer* negative control | N/A | Invitrogen | AM4611 |
| ***MYC*** | MYC siRNA 1 | Stealth siRNA | HSS106837 | Invitrogen | 1299001 |
|  | MYC siRNA 2 | Stealth siRNA | HSS181389 | Invitrogen | 1299001 |
| ***PFKFB3*** | PFKFB3 siRNA 1 | Stealth siRNA | HSS107860 | Invitrogen | 1299001 |
|  | PFKFB3 siRNA 2 | Stealth siRNA | HSS107862 | Invitrogen | 1299001 |

**Supplementary Table 2: List of siRNA.** Comprehensive list of the siRNA utilized in this investigation.

| **Assay**  **(Agilent Catalog No.)** | **Substrate** | **Target** | **Final concentration** |
| --- | --- | --- | --- |
| **Glycolysis stress test**  **(103020-100)** | Glucose | Glycolysis substrate | 10mM |
|  | Oligomycin | ATP synthase inhibitor | 1.0µM |
|  | 2-DG | Glycolysis inhibitor | 50mM |
| **Mito stress test**  **(103015-100)** | Oligomycin | ATP synthase inhibitor | 1.0µM |
|  | FCCP | Mitochondrial membrane uncoupler | 1.0µM |
|  | Rotenone/Antimycin A | ETC Complex I/III inhibitors | 0.5µM/0.5µM |

**Supplementary Table 3: Seahorse extracellular flux reagents.** Assay kit reagents used for the glycolysis stress test and mito stress test during Seahorse extracellular flux analysis.

**Supplementary Figure 1: Metabolic characterization of MYC-expressing tumors and cell lines. A)** There are 364 genes commonly down regulated in the MYC^High^ subset of the George et al, Gay et al, and Sato et al datasets. **B)** 19.7% of the down regulated genes are linked to a metabolic process. **C)** The top GO terms for the down regulated genes are not implicated in metabolic pathways. **D)** *HK2*, *PFKFB3*, and *LDHA* gene expression are decreased in MYC^High^ patient samples. **E)** Proline significantly and positively correlates to *MYC* expression. **F)** Arachidonyl-carnitine significantly and negatively correlates to *MYC* expression. **G)** Metabolites isolated from normal lung tissue and spontaneous RPM lung tumors revealed 57 out of 108 significantly altered metabolites. **H-I)** Lactate and proline are significantly increased in RPM tumors. **J-K)** Pyruvate and carnitine are significantly reduced in RPM tumors.


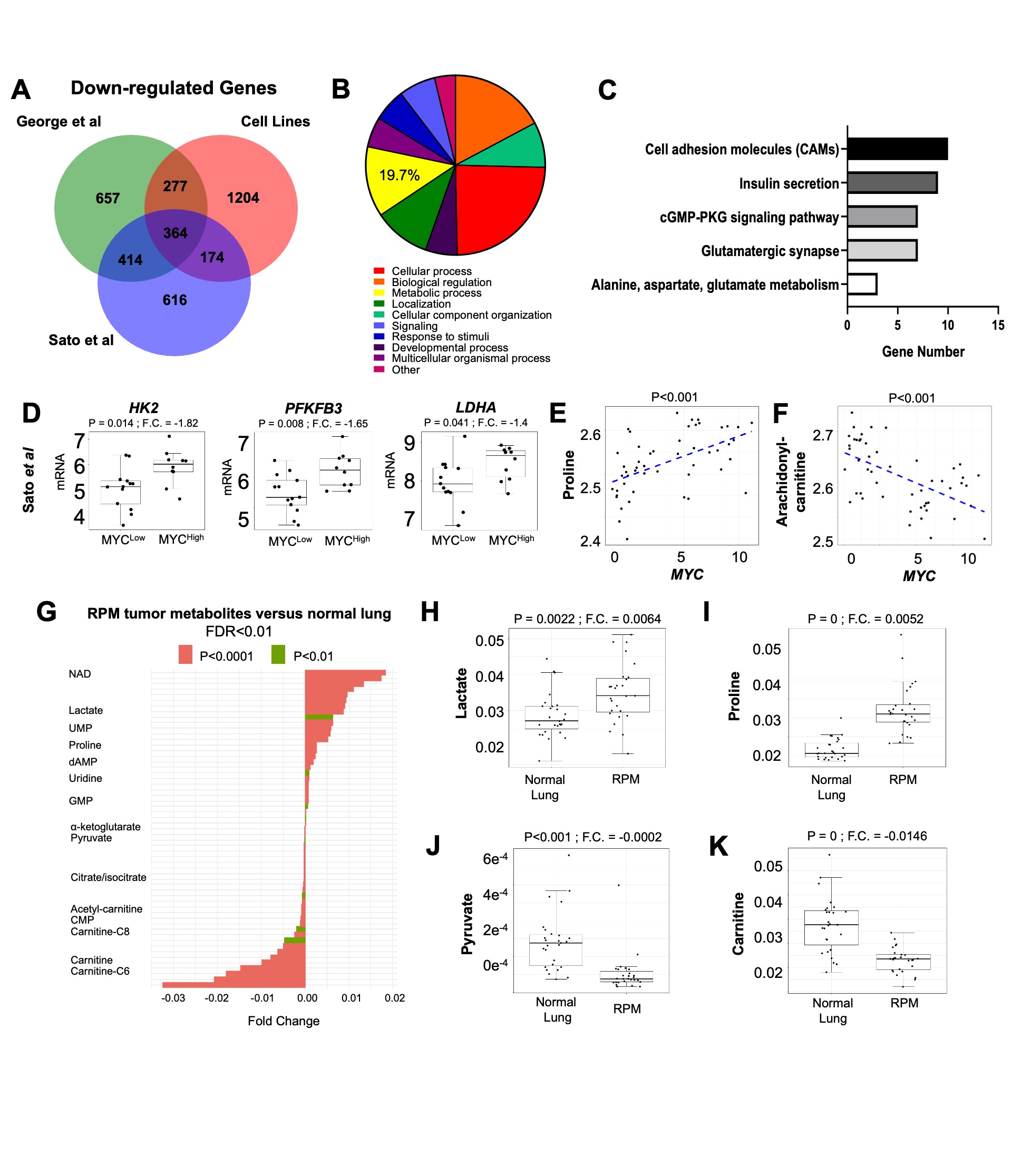


**Supplementary Figure 1: Metabolic characterization of MYC-expressing tumors and cell lines. A)** There are 364 genes commonly down regulated in the MYC^High^ subset of the George et al, Gay et al, and Sato et al datasets [11, 37, 38]. **B)** 19.7% of the down regulated genes are linked to a metabolic process. **C)** The top GO terms for the down regulated genes are not implicated in metabolic pathways. **D)** *HK2*, *PFKFB3*, and *LDHA* gene expression are decreased in MYC^High^ patient samples.[38] **E)** Proline significantly and positively correlates to *MYC* expression.[39] **F)** Arachidonyl-carnitine significantly and negatively correlates to *MYC* expression.[39] **G)** Metabolites isolated from normal lung tissue and spontaneous RPM lung tumors revealed 57 out of 108 significantly altered metabolites. **H-I)** Lactate and proline are significantly increased in RPM tumors. **J-K)** Pyruvate and carnitine are significantly reduced in RPM tumors.


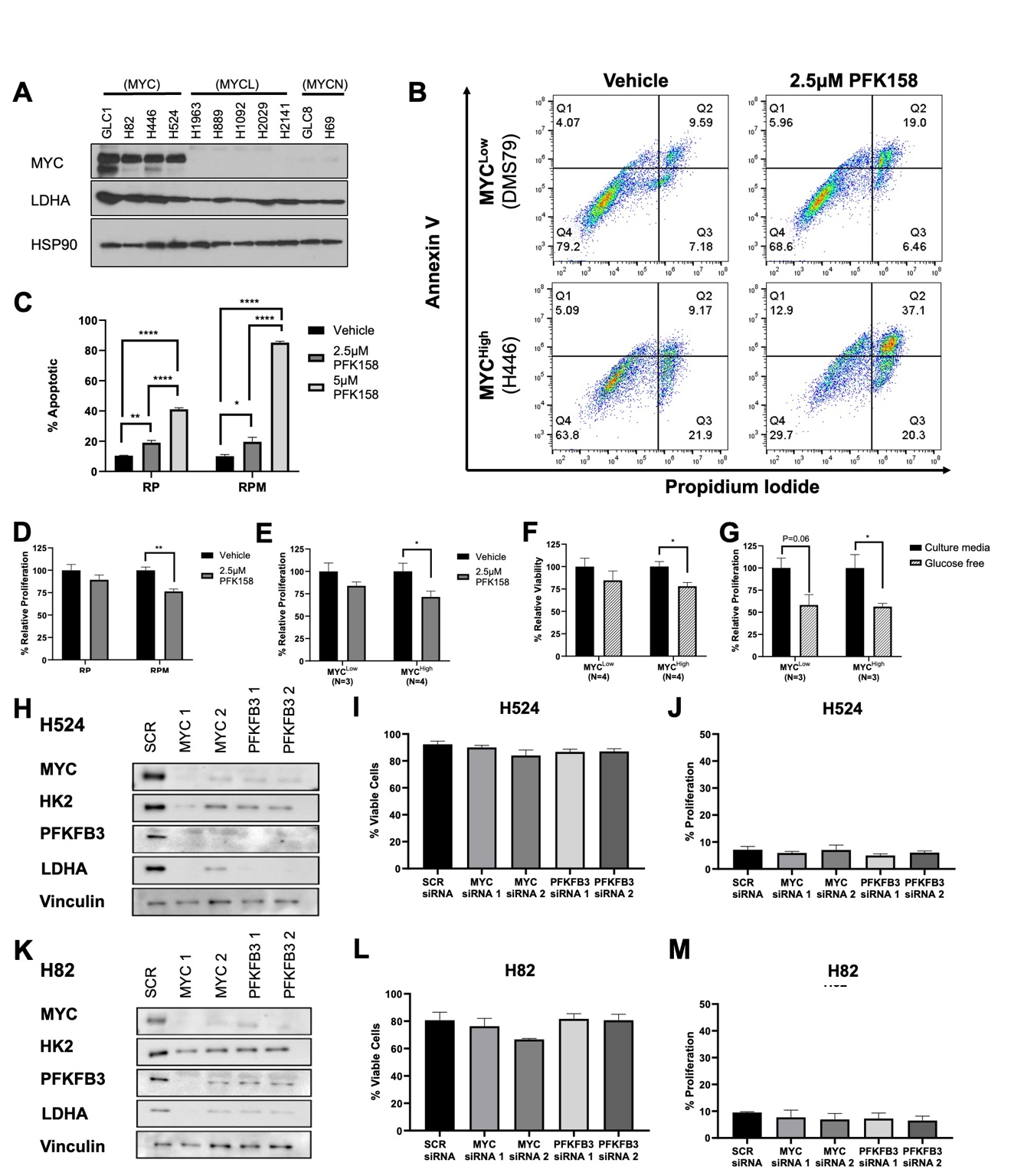


**Supplementary Figure 2: PFK158 and glucose restriction reduce viability and proliferation. A)** Immunoblot of cell lines classified as MYC, MYCL, or MYCN showing protein expression of MYC and LDHA with HSP90 loading control. **B)** Representative MYC^Low^ (DMS79) and MYC^High^ (H446) apoptotic flow plots after treatment with 2.5µM PFK158. **C)** The percent of apoptotic RP and RPM cells increases with higher doses of PFK158. **D)** The percent proliferation of RP and RPM cells is significantly decreased after 2.5µM PFK158 treatment. **E)** The percent proliferation of MYC^Low^ (H2029, DMS79, H526) and MYC^High^ (H847, H146, H82, H446) cells is significantly decreased after 2.5µM PFK158 treatment. **F-G)** Glucose restriction reduces viability and proliferation in MYC^High^ (H146, H446, H847, H865) cell lines but not MYC^Low^ (H526, H2029, DMS79, H1836) cell lines. **H)** Immunoblots of H524 cell line transfected with SCR siRNA, MYC siRNA, and PFKFB3 siRNA showing lower MYC, HK2, PFKFB3, and LDHA protein expression with vinculin loading control. **I)** The percent of viable cells among siRNA treated H524 cells is not significantly altered. J**)** The percent proliferation cells among siRNA treated H524 cells is not significantly altered. **K)** Immunoblots of H82 cell line transfected with SCR siRNA, MYC siRNA, and PFKFB3 siRNA showing lower MYC, HK2, PFKFB3, and LDHA protein expression with vinculin loading control. **L)** The percent of viable cells among siRNA treated H82 cells is not significantly altered. M**)** The percent proliferation cells among siRNA treated H82 cells is not significantly altered. (*P<0.05; **P<0.01; ****P<0.001)


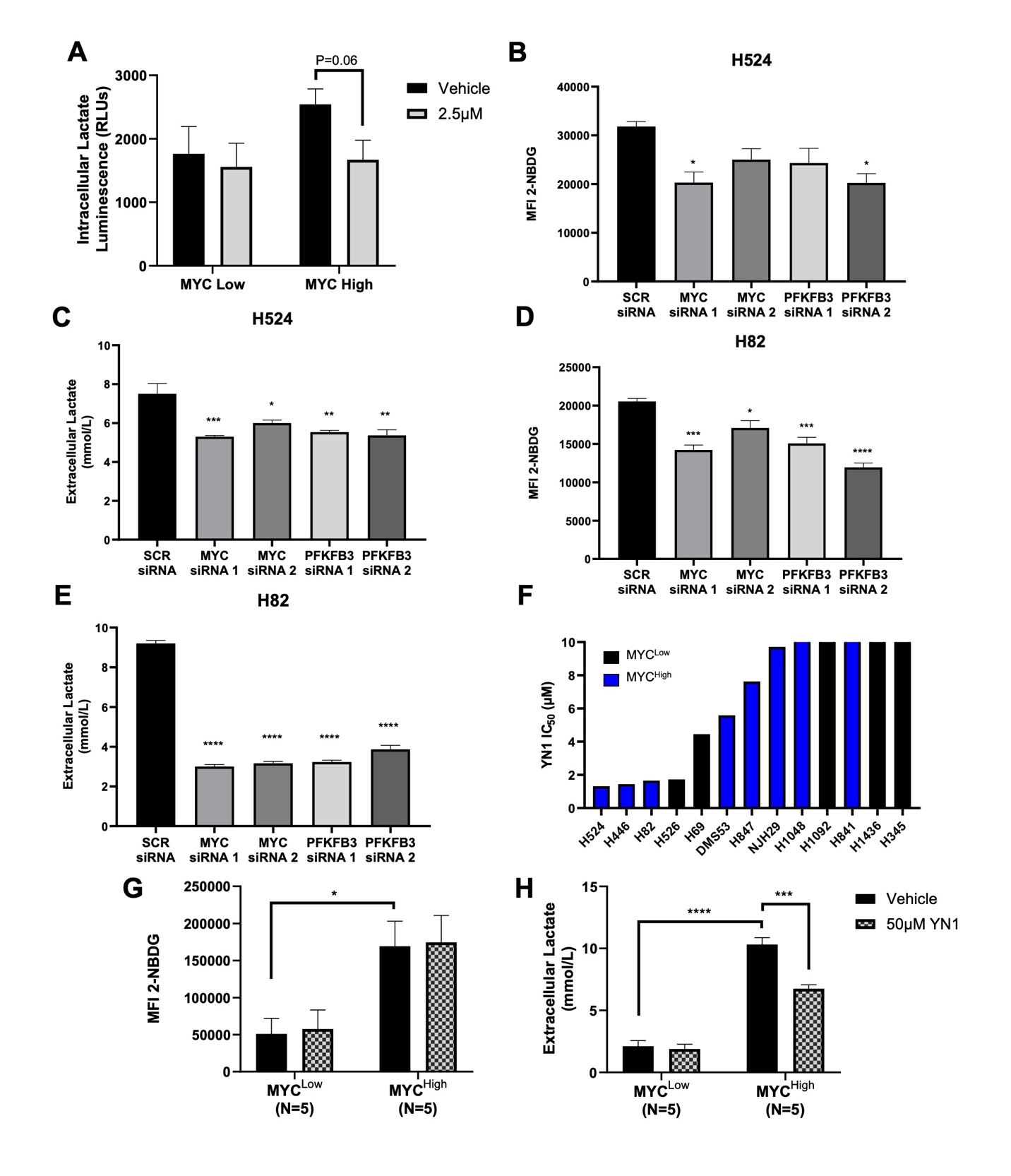


**Supplementary Figure 3: Targeting glycolysis reduces ATP generation and lactate secretion. A)** Intracellular lactate trends downward in MYC^High^ (NJH29, H446, H82, H1930, H1048, H841) and MYC^Low^ (H1092, H1436, DMS79, H526) cells treated with 2.5µM PFK158. **B)** Glucose uptake in significantly lower in H524 cells treated with siRNA against *MYC* and *PFKFB3* compared to a SCR control. **C)** Extracellular lactate is significantly reduced in H524 cells treated with siRNA against *MYC* and *PFKFB3* compared to a SCR control. **D)** Glucose uptake in significantly lower in H82 cells treated with siRNA against *MYC* and *PFKFB3* compared to a SCR control. **E)** Extracellular lactate is significantly reduced in H82 cells treated with siRNA against *MYC* and *PFKFB3* compared to a SCR control. **F)** IC_50_ values of cell lines treated with the glycolysis inhibitor YN1. **G)** There are no significant differences in glucose uptake after YN1 treatment; MYC^Low^ (H1436, DMS79, H1092, H526, H1522) cell lines; MYC^High^ (H446, H82, NJH29, H841, H524) cell lines. **H)** Extracellular lactate is significantly reduced after YN1 treatment in MYC^High^ cell lines; MYC^Low^ (H1436, DMS79, H1092, H526, H1522) cell lines; MYC^High^ (H446, H82, NJH29, H841, H524) cell lines. (*P<0.05; **P<0.01; ***P<0.005; ****P<0.001)


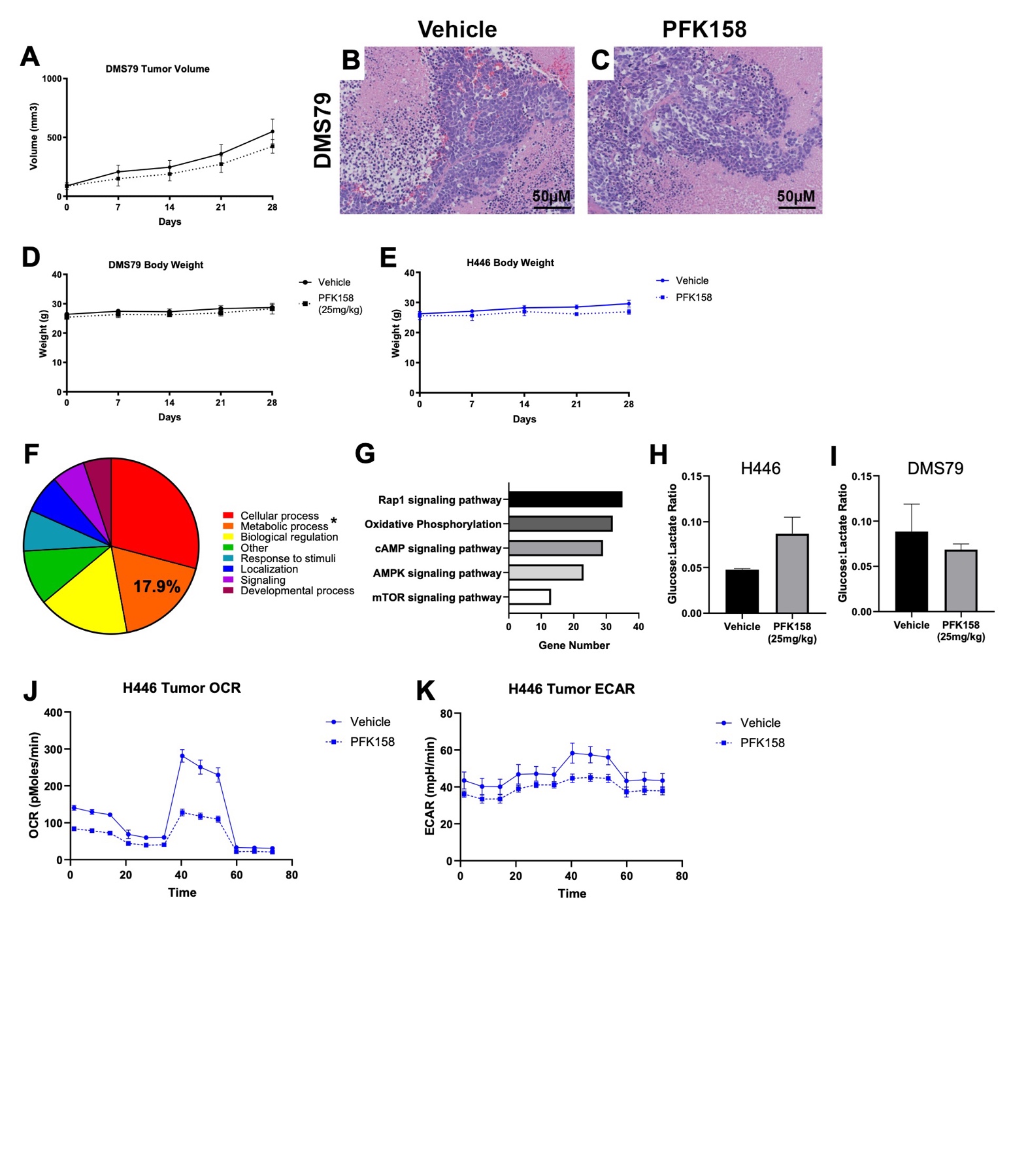


**Supplementary Figure 4: PFK158 treatment in H446 and DMS79 tumors. A)** DMS79 tumor growth curve showing no statistical difference. **B-C)** H&E of DMS79 tumors showing similar pathology. **D-E)** Body weight curves of DMS79 and H446 xenografts with no statistical differences. F**)** 17.9% of genes that are significantly altered between vehicle and PFK148-treated H446 xenografts are linked to a metabolic process. **G)** Several of the top GO terms related to the significantly altered genes between the vehicle and PFK158-treated H446 animals. **H-I)** The glucose-to-lactate metabolite ratio in H446 and DMS79 tumors. **J)** Oxygen consumption rate of H446 xenograft tumors (N=1) treated with vehicle or PFK158 analyzed in six technical replicates. **K)** Extracellular acidification rate of H446 xenograft tumors (N=1) treated with vehicle or PFK158 analyzed in six technical replicates.
